# Priority Setting in HIV, Tuberculosis, and Malaria – New Cost-Effectiveness Results From WHO-CHOICE

**DOI:** 10.34172/ijhpm.2020.251

**Published:** 2021-01-03

**Authors:** Ambinintsoa H. Ralaidovy, Jeremy Addison Lauer, Carel Pretorius, Olivier JT Briët, Edith Patouillard

**Affiliations:** ^1^World Health Organization (WHO), Geneva, Switzerland.; ^2^CERDI-CNRS-IRD-UCA, Clermont-Ferrand, France.; ^3^University of Strathclyde, Glasgow, UK.; ^4^Avenir Health, Glastonbury, CT, USA.; ^5^Swiss Tropical and Public Health Institute, Basel, Switzerland.; ^6^University of Basel, Basel, Switzerland.

**Keywords:** Cost-Effectiveness Analysis, HIV, Tuberculosis, Malaria, Priority Setting, Universal Health Coverage

## Abstract

**Background:** This paper forms part of an update of the World Health Organization Choosing Interventions that are Cost-Effective (WHO-CHOICE) programmes. It provides an assessment of global health system performance during the first decade of the 21st century (2000-2010) with respect to allocative efficiency in HIV, tuberculosis (TB) and malaria control, thereby shining a spotlight on programme development and scale up in these Millennium Development Goal (MDG) priority areas; and examining the cost-effectiveness of selected best-practice interventions and intervention packages commonly in use during that period.

**Methods:** Generalized cost-effectiveness analysis (GCEA) was used to determine the cost-effectiveness of the selected interventions. Impact modelling was performed using the OpenMalaria platform for malaria and using the Goals and TIME (TB Impact Model and Estimates) models in Spectrum for HIV and TB. All health system costs, regardless of payer, were included and reported in international dollars. Health outcomes are estimated and reported as the gain in healthy life years (HLYs) due to the specific intervention or combination. Analysis was restricted to eastern sub-Saharan Africa and Southeast Asia.

**Results:** At the reference year of 2010, commonly used interventions for HIV, TB and malaria were cost-effective, with cost-effectiveness ratios less than I$ 100/HLY saved for virtually all interventions included. HIV, TB and malaria prevention and treatment interventions are highly cost-effective and can be implemented through a phased approach to full coverage to achieve maximum health benefits and contribute to the progressive elimination of these diseases.

**Conclusion:** During the first decade of the 21st century (2000-2010), the global community has done well overall for HIV, TB, and malaria programmes as regards both economic efficiency and programmatic selection criteria. The role of international assistance, financial and technical, arguably was critical to these successes. As the global community now tackles the challenge of universal health coverage, this analysis can reinforce commitment to Sustainable Development Goal targets but also the importance of continued focus on these critical programme areas.

## Background

 The Sustainable Development Goals address universal health coverage in target 3.8.^1,2^ Priority setting in the context of universal health coverage emphasizes three values: improving population health, ensuring equity in access to and quality of services and avoiding impoverishment or underutilization of services as a result of out-of-pocket expenditures.^3,4^ Although health impact is considered to be the defining purpose of the health system, allocative efficiency can be measured with respect to any one of these values, or with respect to all three together, for example using extended cost-effectiveness analysis.

 Here, we adopt generalized cost-effectiveness analysis (GCEA), an approach used by the World Health Organization’s (WHO’s) programme Choosing Interventions that are Cost-Effective (CHOICE), which has been a global leader in cost-effectiveness analysis in global health since 1998. The GCEA approach has the principal advantage of allowing for critical analysis of the package of currently implemented interventions, along with those that may be additionally considered under scaling-up scenarios.

 We propose to provide a quantitative assessment of allocative efficiency within three critical diseases areas during the first decade of this century. This analysis provides a retrospective evaluation of programme development and scale up during this period. HIV, tuberculosis (TB), and malaria are of interest not only because of their prominence in the Millennium Development Goals (MDGs) but also because of the creation of The Global Fund to Fight AIDS, Tuberculosis and Malaria (The Global Fund), which contributed to an unprecedented increase in funding for combatting these infectious diseases.

This paper forms part of an update of the WHO-CHOICE programme and its previous stand-alone analyses of the cost-effectiveness of interventions to combat HIV, TB, or malaria.^5-7^ As in previous work, we focus here on two economically and epidemiologically diverse regions: eastern sub-Saharan Africa and Southeast Asia^8^ in order to have examples of the indicative cost-effectiveness of a common technology set in diverse settings. We stress the word “indicative,” since the analysis is regional and has not been contextualized to particular country settings, as would be done for example for national and subnational decision-making, programme development and priority setting. Rather, we examine how implementation at a macro scale performed relative to global knowledge about best practice during the period 2000-2010.

Given that *Plasmodium falciparum *is the most prevalent malaria parasite in the WHO African Region and that most (56%) cases of *Plasmodium vivax *malaria occur in the WHO South-East Asia Region,^9^ we focus our analysis on *P. falciparum* malaria, HIV and TB for the eastern sub-Saharan Africa region, and on *P. vivax* malaria, HIV and TB for the South-East Asia region. Finally, in light of the Phase III trials of RTS,S vaccine conducted from 2009 to 2014, we also ask how overall performance would have been changed by the addition of RTS,S vaccine to malaria control programmes.

## Key Messages

**Implications for policy makers**

- Country level: Continue to scale up comprehensive HIV, tuberculosis (TB), and malaria programmes.
- Global level: Continue to provide technical and donor assistance for HIV, TB, and malaria programmes.
- Both: Generalize these practices to the rest of the health system.

**Implications for public**

Although more needs to be done, coverage levels are higher in HIV, tuberculosis (TB) and malaria than for other conditions in the regions studied; moreover, overall and on average the right interventions are being implemented. Yet this is not a cause for complacency. Without continued effort, regression to lower levels of epidemic control is inevitable and in some cases is already being witnessed. International collective action, in conjunction with institutions committed to strengthening domestic actors, has made a convincing case as a global public good for HIV, TB and malaria control, demonstrating that international development assistance for health can be transformative when combined with appropriate country support in the form of technical assistance about intervention choice and programme development.

## Methods

The methods and rationale of GCEA used by WHO-CHOICE have been published elsewhere.^10,11^ The principal advantage of GCEA is that it allows for an analysis of the package of currently implemented interventions, along with those that may be considered under alternative or scaling-up scenarios. The cost-effectiveness of interventions is examined first individually against a “null” scenario, a counterfactual scenario in which the effects of all currently implemented interventions are removed, and second as packages of interventions defined as combinations of the most cost-effective individual interventions. To allow for comparison and integration of results in a sectoral analysis, common methods and assumptions are applied for HIV, TB, and malaria. Health outcomes are measured and reported as the gain in healthy life years (HLYs) due to a specific intervention or combination thereof. For the calculation of HLYs, disease weights were obtained from the Global Burden of Disease study, 2010.^12^ For costing, all market-traded health system inputs are costed, regardless of payer (ie, programme costs, service delivery of the intervention, drugs and expendables). As is common, programmes are considered to be implemented for 100-year lifetime horizon using a population model that calculates duration-dependent life-table effects such as healthy life expectancy. A 3% per annum discount rate is applied to costs in all scenarios. HLY are reported both undiscounted and with a 3% per annum discount rate.

The cost-effectiveness of disease-specific sets of regional counterfactual scenarios is assessed against a null comparator (no intervention), along with individual and combined interventions, including seven scenarios for *P. vivax* malaria (Table 1), 14 for *P. falciparum* malaria (Table 2), 12 for HIV (Table 3) and 10 for TB (Table 4). The effects and costs of current (ie, actual) practice were also assessed relative to this baseline. Interventions are analysed at 50%, 80% and 95% coverages; details for the current scenarios can be found in Tables 5, 6, 7 and 8.

**Table 1:** Interventions Included in the Analysis for *Plasmodium vivax* Malaria

**#**	**Scenario Name**	**Description**
1	CMS	Management of severe cases
2	ITN	Insecticide-treated bed nets
3	CMS_ITN	Management of severe cases + Insecticide-treated bed nets
4	CMU_CMS	Management of suspected uncomplicated cases + Management of severe cases
5	CMU_CMS_ITN	Management of suspected uncomplicated cases + Management of severe cases + Insecticide-treated bed nets
6	CMUPQX^*^_CMS	As #4 with primaquine only given to non-G6PDd males
7	CMUPQX^*^_CMS_ITN	As #5 with primaquine only given to non-G6PDd males

Abbreviation: G6PDd, glucose-6-phosphate dehydrogenase (G6PD) deficient. * PQX: G6PDd testing in males, non-deficient males receive primaquine, and all others (G6PDd males and all females) do not receive primaquine.

**Table 2:** Interventions Included in the Analysis for *Plasmodium falciparum* Malaria

**#**	**Scenario Name**	**Description**
1	CMS	Management of severe cases
2	ITN	Insecticide-treated bed nets
3	CMS_ITN	Management of severe cases + Insecticide-treated bed nets
4	CMU_CMS	Management of suspected uncomplicated cases + Management of severe cases
5	CMU_CMS_ITN	Management of suspected uncomplicated cases + Management of severe cases + Insecticide-treated bed nets
6	CMS_RTSS	Management of severe cases + Malaria vaccine with RTS, S
7	ITN_RTSS	Insecticide-treated bed nets + Malaria vaccine with RTS, S
8	CMS_ITN_RTSS	Management of severe cases + Insecticide treated bed nets + Malaria vaccine with RTS,S
9	CMU_CMS_RTSS	Management of suspected uncomplicated cases + Management of severe cases + Malaria vaccine with RTS, S
10	CMU_CMS_ITN_RTSS	Management of suspected uncomplicated cases + Management of severe cases + Insecticide treated bed nets + Malaria vaccine with RTS,S
11	CMU_D*_CMS	As #4, but treatment seeking fever cases RDT tested
12	CMU_D*_CMS_ITN	As #5, but treatment seeking fever cases RDT tested
13	CMU_D*_CMS_RTSS	As #9, but treatment seeking fever cases RDT tested
14	CMU_D*_CMS_ITN_RTSS	As #10, but treatment seeking fever cases RDT tested

Abbreviation: RDT, Malaria rapid diagnostic test. *D: Diagnostics

**Table 3:** Interventions Included in the Analysis for HIV

**#**	**Scenario Name**	**Description**
1	FSW	Female sex workers and clients
2	PWID	People who inject drugs community outreach and peer education
3	MMCO	Mass media communication designed to increase demand and improve use of condoms, and condom provision
4	MSM	Interventions targeting men who have sex with men
5	VMMC	Voluntary medical male circumcision
6	YFIs	Youth focused interventions
7	ART3	HIV testing services + Antiretroviral therapy for all HIV positive adults with CD4 <350, all HIV positive children ≤2 years, children>2 yrs with CD4 <350, pregnant women Option B+
8	ART5	HIV testing services + Antiretroviral therapy for all HIV positive adults with CD4 <500, all HIV positive children ≤2 years, children>2 yrs with CD4 <500, pregnant women Option B+*
9	TasP	HIV testing services + Antiretroviral therapy treatment as prevention for all HIV positive adults, children and PMTCT Option B+
10	CB1	ART3 + MMCO + FSW +PWID + MSM +YFI + Management of sexually transmitted infections + VMMC
11	CB2	ART5 + MMCO + FSW +PWID + MSM +YFI + Management of sexually transmitted infections + VMMC
12	CB3	TASP + MMCO + FSW +PWID + MSM +YFI + Management of sexually transmitted infections + VMMC

Abbreviation: PMTCT, prevention of mother-to-child transmission. *Pregnant women Option B+: lifelong antiretroviral therapy treatment given to HIV positive pregnant women regardless of CD4 count or WHO clinical stage.^37^

**Table 4:** Interventions Included in the Analysis for TB

**#**	**Scenario Name**	**Description**
1	B2	Treatment (FLD + SLD) + Detection (Xpert + X-ray + Culture) + Drug susceptibility testing
2	B2_AX	Treatment (FLD + SLD) + Detection (Xpert + X-ray + Culture) + Drug susceptibility testing + ART prioritization for TB cases
3	B2_AX_PX_PXC	Treatment (FLD + SLD) + Detection (Xpert + X-ray + Culture) + Drug susceptibility testing + ART prioritization for TB cases + Preventive therapy + Preventive therapy for children
4	B2_PX	Treatment (FLD + SLD) + Detection (Xpert + X-ray + Culture) + Drug susceptibility testing + Preventive therapy
5	B2_PXC	Treatment (FLD + SLD) + Detection (Xpert + X-ray + Culture) + Drug susceptibility testing + Preventive therapy for children
6	B1	Treatment (FLD + SLD) + Detection (Smear + X-ray + Culture) + Drug susceptibility testing
7	B1_AX	Treatment (FLD + SLD) + Detection (Smear + X-ray + Culture) + Drug susceptibility testing + ART prioritization for TB cases
8	B1_AX_PX_PXC	Treatment (FLD + SLD) + Detection (Smear + X-ray + Culture) + Drug susceptibility testing + ART prioritization for TB cases + Preventive therapy + Preventive therapy for children
9	B1_PX	Treatment (FLD + SLD) + Detection (Smear + X-ray + Culture) + Drug susceptibility testing + Preventive therapy
10	B1_PXC	Treatment (FLD + SLD) + Detection (Smear + X-ray + Culture) + Drug susceptibility testing + Preventive therapy for children

Abbreviations: ART, antiretroviral therapy; FLD, first line drugs, SLD, second line drugs; TB, tuberculosis.

**Table 5:** Population in Need and Current Coverage for *Plasmodium vivax* Malaria

**Interventions**	**Population in Need**	**Current Coverage (%)**	**References**
CMU	All - age population, men and women living in malaria endemic areas	52	^20,21,25^
ITN		21	
CMS		48	

Abbreviations: ITN, insecticide-treated net; CMS, management of severe cases; CMU, management of suspected uncomplicated cases.

**Table 6:** Population in Need and Current Coverage for *Plasmodium falciparum* Malaria

**Interventions**	**Population in Need**	**Current Coverage (%)**	**References**
CMU	All - age population, men and women living in malaria endemic areas	40	^20,21,26^
ITN		58	
CMS		48	

Abbreviations: ITN, insecticide treated net; CMS, management of severe cases; CMU, management of suspected uncomplicated cases.

**Table 7:** Population in Need and Current Coverage for HIV

**Intervention**	**Population in Need**	**Current Coverage (%) **		**References**
		**Eastern Sub-Saharan Africa**	**Southeast Asia**	
FSWs and clients	FSWs aged 15-49 years	31	67	^38,39^ UNGASS reports, data collected for the UNAIDS global report (analogous to the TB reports) and data collected for the Resource Needs Model exercises
PWID community outreach and peer education	PWIDs aged 15-49 years (male and female)	10	33	
Mass media	Population aged 15-49 years (male and female)	29	31	
Condom provision	Population aged 15-49 years (male and female)	27	28	
MSM	MSM aged 15-49 years	25	28	
VMMC	Population aged 10-19 years (male)	70	-	
YFIs	Population aged 15-49 years (male and female) and STI symptomatic	60	18	
STI management	Population aged 15-49 years (male and female) and STI symptomatic	36	23	
HIV testing services	Population aged 15-49 years (male and female)	23	4	

Abbreviations: FSWs, female sex workers; PWID, people who inject drug; MSM, Men who have sex with men; VMMC, voluntary medical male circumcision; STI, sexually transmitted infection; YFIs, youth focused interventions; UNGASS, United Nations General Assembly Special Session on Drugs; UNAIDS, the Joint United Nations Programme on HIV/AIDS; TB, tuberculosis.

**Table 8:** Population in Need and Current Coverage for TB

**Interventions**	**Population in Need**	**Current Coverage (%)**		**References**
		**Eastern Sub-Saharan Africa**	** Southeast** **Asia**	
Detection		73	65	
Screening	All age population, men and women			Screening rate is a fitting parameter
Algorithm sensitivity and specificity	TB susceptible and active TB cases, as applicable to diagnostic method			^41-43^
Drug sensitivity test	Active new and previously treated TB cases			^46,47^
Treatment	Active TB cases, with diagnosis and linked to care	85	80	^46,47^
ART prioritization	HIV-positive TB cases, not on ART	80	40	^46,47^
Preventive therapy	Ages 15+, men and women, LTBI cases	40	40	^46,47^
Preventive therapy for children	Ages 0-14, LTBI cases	23	50	^46,47^

Abbreviations: ART, antiretroviral therapy; LTBI, latent TB infection; TB, tuberculosis.

The list of interventions is not exhaustive and excluding an intervention does not imply that it is cost ineffective. The term “current” as used here refers to an intervention that represents the average combination of interventions used in typical countries in the relevant geographical area at year 2010. Some of these interventions, however, do not reflect the recommendations of WHO anymore, and up-to-date WHO recommendations can be found in Table 9. Thus, our results are intended to be indicative of average implementation performance relative to global knowledge of best practices at the time, rather than as prescriptive packages intended for countries to implement now. As noted, our principal objective here is to assess and evaluate retrospectively programmatic performance in HIV, TB and malaria control during the first decade of the 21st century. In addition, in the case of malaria, we also assess the potential cost-effectiveness of the RTS,S vaccine in the context of our GCEA framework.

**Table 9:** WHO Recommended Interventions

**Disease**	**Category**	**Intervention**	**References**
HIV	Prevention	Male and female condoms and lubricants	^48^
		Harm reduction for people who inject drugs	^48,49^
		Antiretroviral-based prevention: pre-exposure prophylaxis, post-exposure prophylaxis, prevention of mother-to child transmission, antiretroviral therapy that achieves viral suppression	^48,50^
		Prevention of HIV infection in infants	^48^
		VMMC	^48^
		Injection and blood safety	^48^
		Behaviour change interventions (specific to particular population groups including adolescent girls and young women)	^48^
		Prevention and management of gender-based and sexual violence	^48^
	Testing	HIV testing	^48,51,52^
	Treatment and Care	Expand antiretroviral therapy coverage	^48,50^
		Prevent and manage HIV and TB coinfection	^48^
		Prevent and manage HIV and viral hepatitis coinfection	^48^
		Address other HIV coinfections	^48^
		Prevent and manage HIV drug resistance	^48^
		Provide person-centred chronic care for people living with HIV	^48^
	Comprehensive package for key populations (MSM, PWIDs, people in prisons and other closed settings, sex workers and transgender people)	Comprehensive condom and lubricant programming	^49^
		Harm reduction interventions for substance use (in particular needle and syringe programmes and, opioid substitution therapy and naloxone distribution)	
		Behavioural interventions	
		HIV testing and counselling	
		HIV treatment and care	
		Prevention and management of co-infections and other co-morbidities	
		Sexual and reproductive health interventions	
TB	Prevention	Treatment of LTBI	^53-55^
		Prevention of transmission of *Mycobacterium tuberculosis* through infection prevention and control	
		Vaccination of children with the BCG vaccine	
	Detection	Early diagnosis of TB including universal drug susceptibility testing, and systematic screening of contacts and high risk groups	^53^
		Rapid molecular test: Xpert® MTB/RIF assay (Cepheid, USA)	^55^
		Sputum smear microscopy	^55^
		Culture-based methods	^55^
		First-line LPAs	^55^
		Second-line LPA	^55^
		DST by phenotypic or genotypic methods should be done for all persons with bacteriologically confirmed TB	^53^
	Treatment and Care	Treatment of all people with TB including drug resistant TB, and patient support	^53,55,56^
		Collaborative TB/HIV activities, and management of co-morbidities	^53,55,57^
Malaria	Prevention (core intervention)	ITNs/LLINs or IRS	^58^
		IPTp	^59,60^
		IPTi	^61^
		SMC	^62^
	Testing	RDTs or microscopy	^63^
	Treatment	Treatment of blood-stage infection (for *P. falciparum* and *P. vivax*)	
		Treatment of liver-stage infection (not applicable for *P. falciparum* and includes G6PD testing for confirmed cases of *P. vivax*)	
		Treatment of severe malaria

Abbreviations: WHO, World Health Organization; VMMC, voluntary medical male circumcision; TB, tuberculosis; PWID, people who inject drug; MSM, Men who have sex with men; LTBI, latent TB infection; BCG, Bacillus Calmette–Guérin; LPA, line probe assays; DST, drug-susceptibility testing; ITNs, insecticide-treated nets; LLINs, long lasting insecticidal nets; IRS, indoor residual spraying; IPTp, Intermittent preventive treatment of malaria in pregnancy; IPTi, Intermittent preventive treatment of infants; SMC, seasonal malaria chemoprevention; RDTs, rapid diagnostic tests.

 An expansion path shows the steps in programme expansion that a hypothetical decision-maker could follow when maximizing health. However, in constructing such an expansion path, even when maximization of population health is the goal it is presumably important to consider other factors too, such as the investment by the health system in a set of fixed assets such as equipment, facilities and human resources that cannot be readily redeployed to alternative uses. We therefore present two expansion paths, one, a health-maximizing expansion path that has no constraints apart from the cost-effectiveness of interventions, and, two, a programmatic expansion path that respects the fact that health system resources represent asset-specific investments that cannot be easily redeployed. In other words, while an “expansion path” reflects an indicative optimal path for the expansion of health services, more broadly speaking the concept of optimal also includes criteria related to programme acceptability. For example, bringing a highly cost-effective intervention to full coverage in a given year only to drop it and replace it with a different intervention, requiring different fixed assets, when higher levels of funding are available the following year is a possibility that can be excluded on programmatic grounds due to the large fixed costs associated with changing asset specific investments. If such a case is suggested on cost-effectiveness grounds in the health maximizing expansion path, the programmatic expansion path can be “forced” to adopt early the intervention that will ultimately be optimal at full implementation. This means that, if a particular technology appears on the expansion path at a higher level of coverage, then for the previous steps, we considered only the most cost-effective combination of interventions that also included this particular technology at the same or lower levels of coverage. This procedure allows for trade-offs between cost-effectiveness and investment in fixed assets (ie, health system strengthening) to be quantified. Finally, we note that the concept of an expansion path in either of these guises (health maximizing or programmatic) is at base only an indicative device to illustrate trade-offs potentially made by the policy-maker in the course of health system development.

These WHO-CHOICE results are provided at regional level; as further contextualization would be necessary for individual country-level implementation,^13^ so these scenarios should be considered only as estimates of actual average performance at macro level, versus the counterfactual of idealized average performance at macro level, during the period 2000–2010.

###  Impact Modelling

####  Malaria Model

Simulations for *P. falciparum* malaria and *P. vivax* malaria were performed using the OpenMalaria platform,^14^ an open-source C++ program for micro-simulating malaria epidemiology and the impacts of interventions on disease burden. A WHO-CHOICE population model, PopMod,^15^ was used to combine projected case incidence, parasite clearance and mortality data from OpenMalaria with health state valuations in order to calculate the population impact of the scenarios.

All malaria simulations were based on a scenario used earlier.^16-18^ A major innovation compared to that scenario^16^ is the inclusion of fevers with non-malarial aetiology. This scenario including non-malarial fever modelling was adapted to country-specific conditions for: seasonality of transmission, history of insecticide-treated net (ITN) use, history of case management coverage and intensity of transmission. For *P. vivax*, the prevalence of G6PD deficiency^19^ was also taken into account.

Management of severe cases was presumed to be constant over time and across countries of the same region, and the probability of treatment per five-day time step was assumed to be 48%.^20,21^ The per-capita rates of malaria cases and deaths from OpenMalaria were scaled to WHO case incidence estimates per country in 2010.^22^ Similarly, the number of treatments (with or without diagnostic tests), and the number of diagnostic tests, at a given coverage level were also scaled to the WHO estimates of cases.

####  Interventions Against Malaria

**ITNs.** Net distribution was modelled on an annual basis and their effect was hypothesised to last one year (modelled with a step-wise attrition function) in order to prevent periodic effects that complicates the analysis without adding information about intervention efficiency. During the year, no chemical or physical decay was modelled. Costing of nets, however, was done on the basis of actual estimates of useful life.^23^ For *P. falciparum* malaria, ITN efficacy was based on *Anopheles gambiae,*^16-18^ while for *P. vivax* malaria, it was based on *Anopheles epiroticus.*^16-18^

**Case management and diagnostic testing. **Without testing, fevers without parasites, fevers with incidental parasites (ie, fevers that occur in people that are not caused by the malaria infection), and malarial fevers (ie, fevers caused by the malaria infection) have an equal probability of being treated with an antimalarial. With testing, fevers without parasites are not treated. Without G6PD testing, all *P. vivax* positive patients except pregnant women should receive primaquine. With G6PD testing, a status of non-eligibility for primaquine (either due to G6PD deficiency, or due to policy regarding primaquine treatment) was assigned at birth with a pre-set probability dependent on the proportion of hemizygous men in the population.

**RTS,S.** Malaria vaccine was modelled as previously done by Swiss TPH for the Malaria Vaccine Initiative.^24^ While this vaccine is not yet recommended for wide deployment by WHO, it is a potential new intervention that was included in this study on account of the persistent policy interest it attracts.

####  HIV Model

Simulations for HIV were performed with the Goals model, a dynamic compartmental model developed in the Spectrum suite of models.^27-32^ The Goals model is widely-used to produce projections of epidemic trends as well as projections of the impact of interventions. It has been used in many regions, particularly in the Southern and Eastern African region, to study the cost and impact of national and other HIV strategies.

 Goals simulates transmission of HIV and its morbidity and mortality consequences for adult populations aged 15–49 years, which are structured into five risk categories: stable couples (men and women reporting a single partner in the last year), multiple partners (men and women with more than one partner in the last year), female sex workers (FSW) and clients, men who have sex with men (MSM), and people who inject drugs (PWID). These groups are based on risk stratifications available in publicly available data sources, such as Demographic and Health Surveys and AIDS Indicator Surveys, as well in behavioural surveys. HIV transmission in Goals is explicitly calculated from behavioural (eg, age at first sex, number of sexual partners and number of sex acts per sexual partner) and biomedical (eg, antiretroviral therapy [ART], condom use and voluntary medical male circumcision [VMMC]) characteristics.

Goals is directly linked to the AIDS Impact Model (AIM) module in Spectrum, which is used annually to produce national HIV burden estimates towards the Global AIDS report.^28,29^ Goals uses the HIV progression structure in AIM, in which HIV progression is captured through movement between CD4 categories, which form the basis of ART eligibility criteria, ART initiation and ART coverage levels and is also the basis of mortality patterns.

AIM also estimates the effects of programs preventing mother-to-child transmission.^33,34^ AIM further calculates corresponding epidemic patterns for children (0–14 years) and models HIV progression for adults above 49 years.

####  Interventions Against HIV

The impact of behavioural interventions for HIV is represented by an impact matrix which summarizes the impact of key behavioural interventions (eg, community mobilization, mass media campaigns, condom distribution programs, outreach to key populations) with respect to the reduction of condom non-use, reduction of number of partners, and increase in age at first sex for the populations outlined above, based on meta-analyses of research studies.^33-36^

 In addition to these behavioural factors, HIV transmission risk further depends on biomedical factors including ART use, VMMC, the prevalence of other sexually transmitted infections (STIs) and the use of pre-exposure prophylaxis.

 Interventions in Goals can change any of these factors, and thereby affect HIV transmission risk and the future course of the epidemic.

 To apply intervention structure of the Goals model to our CHOICE scenarios, we constructed three ART scenarios in which eligibility for ART is progressively relaxed. In the first scenario ART is provided to all children 2 years and younger, to all other children under the age of 15, to all adults (15 years and older) with CD4 count below 350 cells/uL and Option B+ (ART continued after a pregnancy during which ART is initiated) is followed in the PMTCT (prevention of mother-to-child transmission) program. The second scenario is the same except that a CD4 count below 500 cells/uL replaces CD4 350 cells/uL in the definition of the first scenario. In the third scenario CD4 count is removed as an eligibility criterion and ART is applied as prevention (the so-called TasP strategy). All these strategies assume HIV testing services as part of ART enrolment process. Testing is an entry point and it matters who gets testing services since impact depends on it.

 The list of interventions is extended through VMMC, STI treatment, behavioural interventions (mass media, condom distribution and youth-based programs) as well as outreach programs to high-risk groups (FSW and their clients, PWID and MSM outreach). Three combination scenarios are defined by adding these interventions to the three ART scenarios.

####  Tuberculosis Model

Simulations for TB were performed with the Impact component of the TB Impact Model and Estimates (TIME) model, a dynamic compartmental TB model developed in the open-source Spectrum suite of models.^27,40^

 TIME is used by TB policy-makers and national TB programmes to develop strategic responses and strategies for TB and to produce projections that inform funding applications. The model has been used in most TB settings, including in countries where TB is driven by HIV, in weak health systems, in countries with high MDR-burden and in countries where TB programs depend on a high level of private-sector involvement. The Estimates component of TIME was used by the Global TB Programme to produce estimates for HIV-TB burden towards the Global TB Report.

 The TIME model reflects key aspects of the natural history of TB including primary and latent infection, re-infection and re-activation of latent TB. Smear positivity, negativity and smear conversion is explicitly handled. TIME also accounts for the characteristics of paediatric TB, treatment history and drug resistance. It has additional structure for HIV/ART which mimics the structure of the Spectrum AIM module to use its HIV programmatic data directly. TIME includes two generic TB strains: susceptible and resistant to multi-drug treatment. Resistance can be acquired during treatment upon transmission, at rates that distinguish it from the susceptible TB type in the model.

####  Interventions Against Tuberculosis

A description of the TIME model and its parametrization can be found in the technical appendix of Houben et al.^40^ Interventions in TIME are structured according to a general care-and-control cascade for TB, which is further structured by HIV and MDR status as relevant. The cascade starts with a screening rate which is defined for smear-positive cases, and relative screening rates are specified for smear-negative and TB susceptible cases. Diagnosis of TB is defined by sensitivity and specificity values which are used to characterise the most widely-used and WHO-recommended diagnostic tools in diagnostic pathways for TB. Estimates of diagnostic sensitivity and specificity used in TIME are based on those discussed in.^41-43^

 Following screening and diagnosis, cases are linked to care at a specified acquisition rate and then treated at a specified success rate. The model does not explicitly model a delay between diagnosis and treatment. Coverage, sensitivity and specificity of drug-susceptibility testing (DST) for treatment naïve and previously treated cases are specified. These inputs characterize MDR diagnosis and notification, including notification of non-MDR cases as MDR due to non-perfect specificity of DST.

 The model has a detailed structure for active case finding and household-based contact tracing for children and adults as well as subsequent links to preventive therapy for cases identified with latent TB on the basis of a detailed testing algorithm. Prioritized access to ART for HIV-positive TB cases is explicitly linked to ART enrolment numbers from the Spectrum AIM model.

 To use the intervention structure of the TIME model in our CHOICE analysis, we constructed a basic care-and-control scenario which comprise screening (of all populations, including smear, HIV and MDR-status), detection (including DST to find MDR among new and retreatment cases) and treatment for non-MDR and MDR case (including cases that are false diagnosed due to non-perfect specificity). The different components of the basic care cascade cannot be individually studied, but rather only as packages.

 The basic package, however, has two variations. One represents a traditional diagnostic algorithm of symptomatic screening, followed with smear microscopy or clinical diagnosis and culture for MDR diagnosis. A second scenario represents a recommended design for future diagnostic algorithms which are projected to change to an increasing and dominant use of X-ray for screening and rapid molecular tests such GeneXpert for diagnosis of non-MDR and detection of rifampicin resistance, and by assumption diagnosis of the general MDR strain in our model.

Core interventions recommended in the End TB Strategy^44^ and the Global Plan to End TB 2016-2020^45^ are added to the basic care-and-control cascade. First is preventive therapy for HIV-positive TB cases not on ART and on ART with latent TB infection (LTBI). Then preventive therapy for children ages (0-14) with LTBI found in the context of house-hold screening of index cases. Finally, we added ART prioritization for notified HIV-positive TB cases, irrespective of CD4 count.

 This overall intervention structure is kept general and does not address specific activities or implementation approaches that are necessary to implement the package. In different TB contexts screening rates might be increased through active case finding and enhanced passive case finding in specific groups at high risk of TB infection (eg, diabetics, prisoners, miners, and so on). Community health workers are often employed to improve high treatment success. We made no assumptions regarding these types of underlying activities that are required to achieve the coverage levels of the intervention packages studied.

 We also made no assumption regarding the future trend of the number of tests that will be needed to find one case, and kept the value fixed at 10, which is considered an average value. Generally, it is expected that this value will increase as more aggressive screening policies are adopted by national TB programmes. These are considered too context specific to study here.

###  Interventions Costs

We used a framework developed for WHO-CHOICE for costing interventions. This framework includes patient-level delivery costs, programme costs, and other health system costs, regardless of payer (eg, private or public). We developed the costing estimates under the assumption that health system capacity is available to support the interventions. The quantities of resources assumed used at patient level were based on adherence to WHO treatment guidelines. Programme costs were calculated in a standardized way, as reported in.^64^ Costs were discounted at 3% per annum, and capital expenses annualized over the lifetime of the good. All prices are reported in 2010 international dollars. Costing details for each programme area can be found in Tables 10, 11, 12 and 13.

**Table 10:** Intervention Costing Assumptions for *Plasmodium vivax* Malaria

Patient Costs (*Regional Average Unit Costs Per Person Per Year – I$ 2010)
Management of uncomplicated cases	6.33
Management of severe cases	173.91
ITNs	5.47

Abbreviation: ITNs, insecticide-treated nets. * Prices of drugs from various sources: MSH database: http://erc.msh.org/, UNICEF LLIN data (2014). UNICEF supply catalogue (2012) and WHO-CHOICE price database. Unit costs include logistics, wastage, and freight and insurance (when relevant). ITN’s unit cost is the average cost per net delivered. Consumables required for management for severe cases include those needed for pre-referral treatment, hospital treatment, and post-discharge follow-up.

**Table 11:** Intervention Costing Assumptions for *Plasmodium falciparum * Malaria

Patient Costs (*Regional Average Unit Costs Per Person Per Year - I$ 2010)	
Management of suspected uncomplicated cases	2.06
Management of suspected uncomplicated cases (without diagnosis)	1.41
Management of severe cases	57.55
ITNs	5.47
RTS,S	7.25

Abbreviation: ITNs, insecticide-treated nets. * Prices of drugs from various sources: MSH database: http://erc.msh.org/, UNICEF LLIN data (2014). UNICEF supply catalogue (2012), WHO/IVB/06.15^65^ and WHO-CHOICE price database. Unit costs include logistics, wastage, and freight and insurance. ITN’s unit cost is the average cost per net delivered. RTS,S’s unit cost is the average cost per dose delivered. Consumables required for management for severe cases include those needed for pre-referral treatment, hospital treatment, and post-discharge follow-up.discharge follow-up.

**Table 12:** Intervention Costing Assumptions for HIV

**Patient Costs (I$ 2010) (regional Average Unit Costs Per Person Reached Per Year)**	**Eastern Sub-Saharan Africa**	**Southeast Asia**
Youth-focused interventions	12.22	13.97
FSWs and clients	8.78	28.29
MSM	9.30	32.82
IDU community outreach and peer education	6.60	21.80
Condom provision	0.50	0.15
STI management	9.19	34.07
Voluntary counselling and testing	12.48	31.73
VMMC	56.26	58.11
PMTCT screening	4.43	3.74
PMTCT ARVs	597.23	986.63
Mass media	0.95	0.95
Service Delivery	100.77	54.95
ART		
Labs	33.23	259.50
ARVs - 1st line	130.35	128.65
ARVs - 2nd line	308.34	518.33
Pre-ART	118.74	233.93
Non-ART care and prophylaxis	302.70	302.70
Palliative care	236.14	236.14

Abbreviations: FSWs, female sex workers; MSM, Men who have sex with men; IDU, injecting drug users; STI, sexually transmitted infection; VMMC, voluntary medical male circumcision; PMTCT, prevention of mother-to-child transmission; ARVs, antiretroviral; ART, Antiretroviral therapy.

**Table 13:** Intervention Costing Assumptions for TB

**Patient Costs (I$ 2010) (Regional Average Unit Costs Per Person Reached Per Year)**	**Eastern Sub-Saharan Africa**	**Southeast Asia**
**Detection**		
**Smear microscopy**		
Diagnostic test for passive TB case finding, BAC+ cases	1.20	1.20
Diagnostic tests for adults in HH-contact tracing	1.20	1.20
Diagnostic tests for children in HH-contact tracing	1.20	1.20
Diagnostic test for retreatment cases	1.20	1.20
Diagnostic test for child cases, BAC+ cases	1.20	1.20
Diagnostic test for HIV+ cases, BAC+ cases	1.20	1.20
Test to monitor treatment for new cases	1.20	1.20
Test to monitor treatment for retreatment cases	1.80	1.80
Test to monitor treatment for MDR-TB cases	7.21	7.21
**Culture**		
Diagnostic test for passive TB case finding, BAC+ cases	10.59	10.59
Diagnostic tests for adults in HH-contact tracing	10.59	10.59
Diagnostic tests for children in HH-contact tracing	10.59	10.59
Diagnostic test for smear negative or Xpert negative	10.59	10.59
Diagnostic test for extra pulmonary TB	10.59	10.59
Diagnostic test for child cases, BAC+ cases	10.59	10.59
Diagnostic test for HIV+ cases, BAC+ cases	10.59	10.59
Test to monitor treatment for new cases	21.17	21.17
Test to monitor treatment for retreatment cases	31.76	31.76
Resistance testing for new cases (FLD)	10.59	10.59
Resistance testing for retreatment cases (FLD)	10.59	10.59
Resistance testing/monitoring for MDR-TB cases (FLD)	127.03	127.03
Resistance testing/monitoring for MDR-TB cases (SLD)	127.03	127.03
Resistance testing for HIV+ cases	10.59	10.59
Resistance testing for child cases	10.59	10.59
Resistance testing for MDR-TB contact tracing	10.59	10.59
**Molecular: Xpert**		
Diagnostic test for passive TB case finding, BAC+ cases	9.98	9.98
Diagnostic tests for adults in HH-contact tracing	9.98	9.98
Diagnostic tests for children in HH-contact tracing	9.98	9.98
Diagnostic test for smear negative TB	9.98	9.98
Diagnostic test for extra pulmonary TB	9.98	9.98
Diagnostic test for child cases, BAC+ cases	9.98	9.98
Diagnostic test for HIV+ cases, BAC+ cases	9.98	9.98
Test to monitor treatment for new cases	19.96	19.96
Test to monitor treatment for retreatment cases	29.94	29.94
Resistance testing for new cases	9.98	9.98
Resistance testing for retreatment cases	9.98	9.98
Resistance testing for MDR-TB cases	119.76	119.76
Resistance testing for HIV+ cases	9.98	9.98
Resistance testing for child cases	9.98	9.98
Resistance testing for MDR-TB contact tracing	9.98	9.98
**X-rays, Full Chest**		
Screening for passive TB case finding, BAC+ cases	10.00	10.00
Diagnostic tests for adults in HH-contact tracing	10.00	10.00
Diagnostic tests for children in HH-contact tracing	10.00	10.00
Screening for smear negative TB	10.00	10.00
Screening for extra pulmonary TB	10.00	10.00
Screening for child cases, BAC+ cases	10.00	10.00
Screening for HIV+ cases, BAC+ cases	10.00	10.00
Test to monitor treatment for new cases	20.00	20.00
Test to monitor treatment for retreatment cases	30.00	30.00
Test to monitor treatment for MDR-TB cases	30.00	30.00
**Treatment**		
**First line treatment**		
First-line TB drugs, initial treatment (adults)	30.93	30.93
First-line TB drugs, initial treatment (children)	24.12	24.12
First-line TB drugs, retreatment	98.90	98.90
**MDR and XDR TB**		
Second-line TB drugs	1866.24	1866.24
XDR treatment	7602.00	7602.00
MDR-adverse events and palliative care	120.00	120.00
XDR-adverse events and palliative care	120.00	120.00
**Collaborative TB and HIV/AIDS interventions**		
HIV testing and counselling	4.80	4.80
Prioritization of ART for TB-HIV co-infected	110.00	110.00
Isoniazid preventive therapy for adults and children with HIV and on ART without TB	5.43	5.43
Isoniazid preventive therapy for adults and children with HIV and not on ART without TB	5.43	5.43
**Preventive therapy for adults and children through HH-contact tracing**		
Preventive therapy for children without TB	5.43	5.43
Preventive therapy for adults without TB	5.43	5.43
**MDR case management**		
MDR case management	11886.99	7135.00
**Health systems costs**		
First line: Hospitalization and Ambulatory Care	23.69	55.66
Second line: Hospitalization and Ambulatory Care	1366.60	2854.58

Abbreviations: TB, tuberculosis; HH, household; BAC, blood alcohol content; FLD, first line drugs, SLD, second line drugs; MDR, multidrug-resistant; XDR, extensively drug-resistant; ART, antiretroviral therapy.

## Results

**Figure 1:**
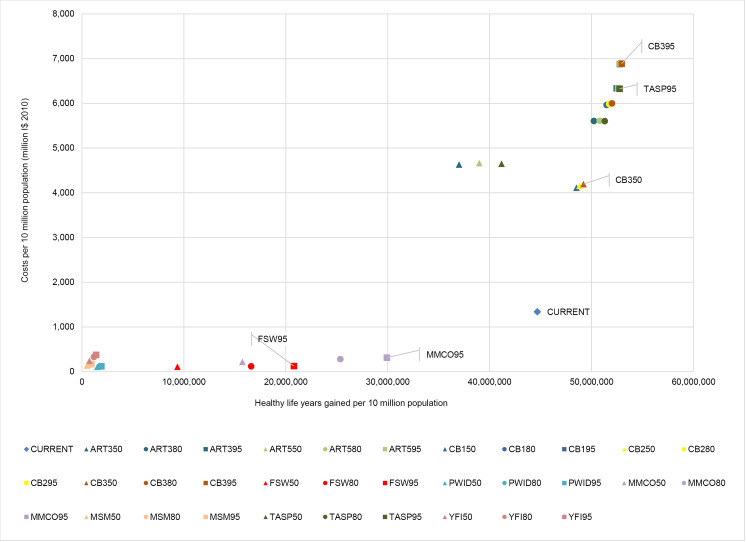
Cost-Effectiveness Expansion Path for HIV Interventions in Southeast Asia. *Refer to Table 1 for intervention label *50, 80 and 95 at the end of each label refer to coverages of 50%, 80% and 95% respectively.

**Figure 2:**
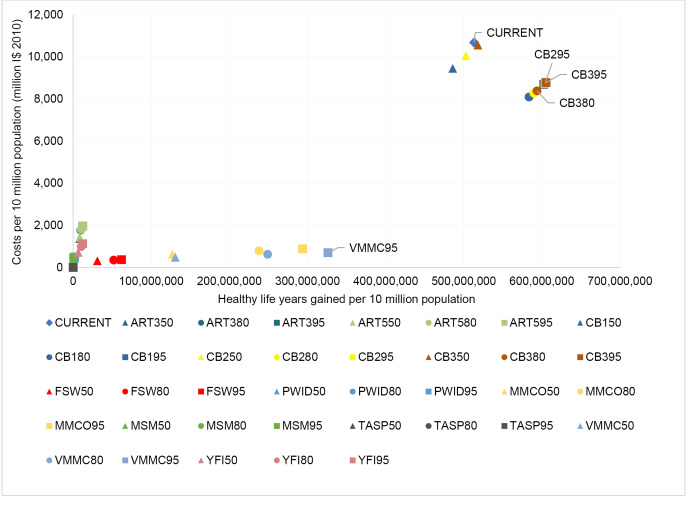
Cost-Effectiveness Expansion Path for HIV Interventions in Eastern Sub-Saharan Africa. *Refer to Table 1 for interventions label *50, 80 and 95 at the end of each label refer to coverage of 50%, 80% and 95% respectively. Abbreviations: ART, antiretroviral therapy; HLY, healthy life year; TasP, Treatment as prevention; MMCO, mass media communication and condom provision; FSW, female sex worker; PWID, people who inject drug; MSM, Men who have sex with men; VMMC, voluntary medical male circumcision; YFI, youth focused intervention.

**Figure 3:**
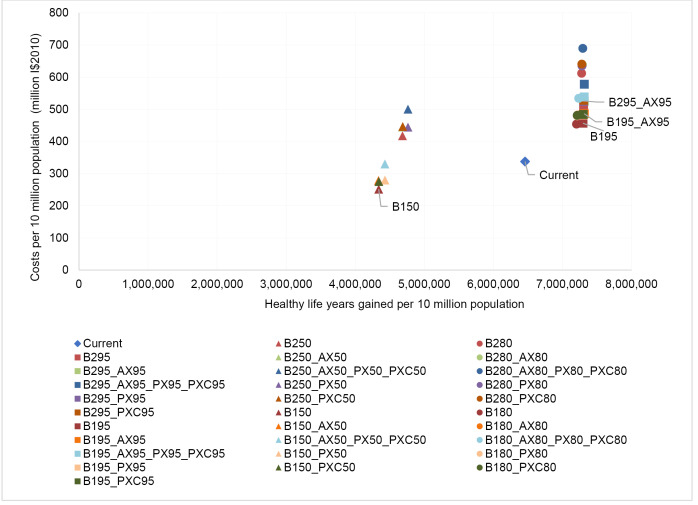
Cost-Effectiveness Expansion Path for TB Interventions in Southeast Asia. * Refer to Table 2 for interventions label *50, 80 and 95 at the end of each label refer to coverage of 50%, 80% and 95% respectively.

**Figure 4:**
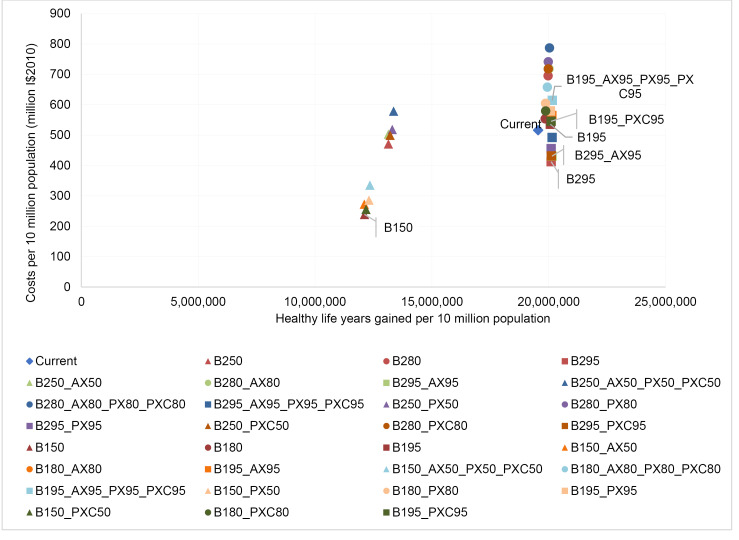
Cost-Effectiveness Expansion Path for TB Interventions in Eastern Sub-Saharan Africa. *Refer to Table 2 for interventions label *50, 80 and 95 at the end of each label refer to coverage of 50%, 80% and 95% respectively.

**Figure 5:**
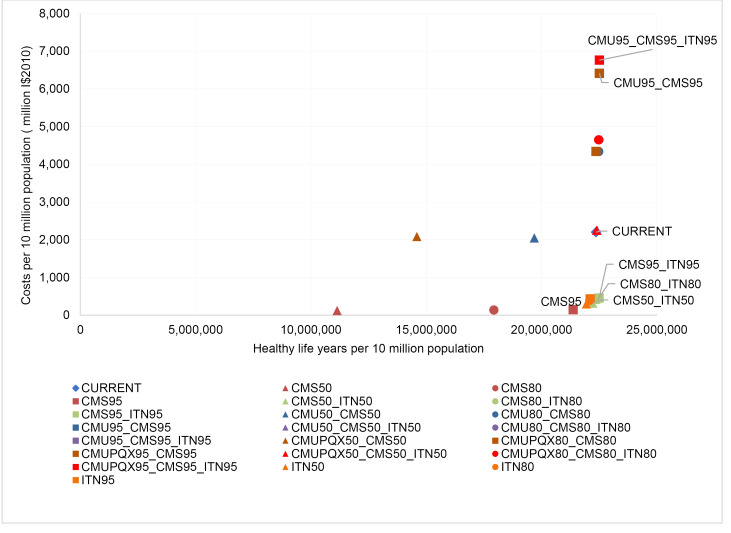
Cost-Effectiveness Expansion Path for Plasmodium * vivax *Malaria Interventions in Southeast Asia. *Refer to Table 5 for interventions label *50, 80 and 95 at the end of each label refer to coverage of 50%, 80% and 95% respectively. Abbreviation: ITN, insecticide-treated net.

**Figure 6:**
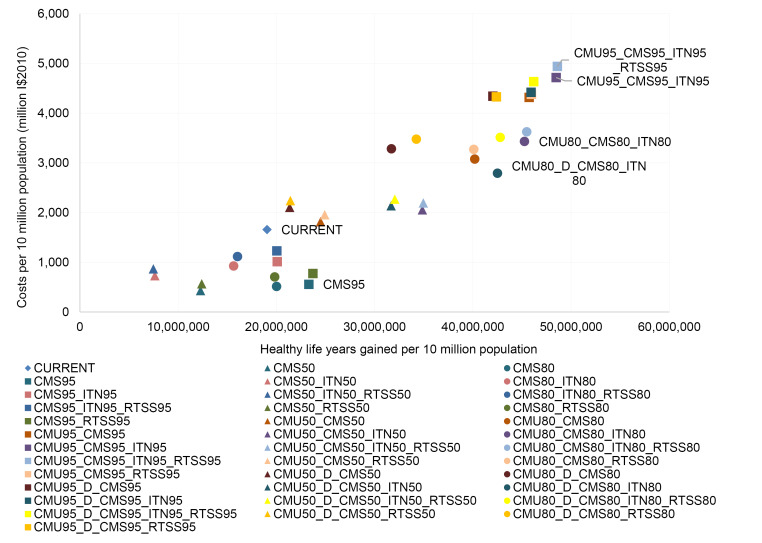
Cost-Effectiveness Expansion Path for P. falciparum Malaria Interventions in Eastern Sub-Saharan Africa. *Refer to Table 6 for interventions label *50, 80 and 95 at the end of each label refer to coverage of 50%, 80% and 95% respectively.

Tables 14-19 show the costs, effects and cost-effectiveness of the different interventions. These tables present only the most cost-effective interventions on the two expansion paths for each of the disease areas. Interventions that are “dominated” ie, are more costly or less effective, are presented in Supplementary file 1. Figures 1-6 show the steps reflecting the expansion paths that a hypothetical decision-maker could follow for the expansion of services with increasing levels of budget. Both the health-maximizing and the programmatic expansion path are presented. However, we consider the programmatic expansion path for the main results, while also discussing where relevant the implications of the health maximizing expansion path. A zoom-in on the high-impact scenarios from the cost-effectiveness expansion path figures can be found in Supplementary file 2.

**Table 14:** Costs, Effects and Cost-Effectiveness of HIV Interventions in Southeast Asia Over 100 Years

	**Intervention**	**Population Coverage (%)**	**Total Costs Per 10 Million Population (Million I$ 2010)**	**HLYs (million HLY) Gained Per 10 Million Population**	**ACER (I$ Per HLY)**	**ICER (I$ Per HLY) (Programmatic expansion path)**	**ICER (I$ Per HLY) (Health Maximizing Expansion Path)**
Current	Current scenario		1339.1	44.7	30		
CB350	TasP + MMCO + FSW + PWID + MSM + YFI + Management of STIs + VMMC	50	4193.6	49.2	85	Dominated	201.1
CB395	TasP + MMCO + FSW + PWID + MSM + YFI + Management of STIs + VMMC	95	6887.5	53.0	130	210.3	2781.0
FSW95	Female sex workers	95	124.3	20.8	6	6.0	6.0
MMCO95	Mass media communication designed to increase demand and improve use of condoms, and condom provision	95	313.4	29.9	10	Dominated	20.7
TASP95	HIV testing services + Antiretroviral therapy treatment as prevention for all HIV positive adults, children and PMTCT Option B +	95	6325.8	52.8	120	Dominated	602.5

Abbreviations: HLY, healthy life year; TasP, Treatment as prevention; MMCO, mass media communication and condom provision; FSW, female sex worker; PWID, people who inject drug; MSM, Men who have sex with men; VMMC, voluntary medical male circumcision; YFI, youth focused intervention; PMTCT, prevention of mother-to-child transmission; STI, sexually transmitted infection; ACER, average cost-effectiveness ratio; ICER, incremental cost-effectiveness ratio.

**Table 15:** Costs, Effects and Cost-Effectiveness of HIV Interventions in Eastern Sub-Saharan Africa Over 100 Years

	**Intervention**	**Population Coverage (%)**	**Total Costs Per 10 Million Population (Million I$ 2010)**	**HLYs (Million Hly ) Gained Per 10 Million Population**	**ACER (I$ Per HLY)**	**ICER (I$ Per HLY) (Programmatic Expansion Path)**	**ICER (I$ per HLY) (Health Maximizing Expansion Path)**
Current	Current Scenario		10682	513	21		
CB295	ART5 + MMCO + FSW + PWID + MSM + YFI + Management of Sexually Transmitted Infections + VMMC	95	8745	604	14	28.9	Dominated
CB380	TasP + MMCO + FSW + PWID + MSM + YFI + Management of STIs + VMMC	80	8386	594	14	Dominated	28.7
CB395	TasP + MMCO + FSW + PWID + MSM + YFI + Management of STIs + VMMC	95	8781	605	15	Dominated	34.2
VMMC95	Voluntary medical male circumcision	95	704	326	2	2.2	2.2

Abbreviations: ART, antiretroviral therapy; HLY, healthy life year; TasP, Treatment as prevention; MMCO, mass media communication and condom provision; FSW, female sex worker; PWID, people who inject drug; MSM, Men who have sex with men; VMMC, voluntary medical male circumcision; YFI, youth focused intervention; STI, sexually transmitted infection; ACER, average cost-effectiveness ratio; ICER, incremental cost-effectiveness ratio.

**Table 16:** Costs, Effects and Cost-Effectiveness of TB Interventions in Southeast Asia Over 100 Years

	**Intervention**	**Population Coverage (%)**	**Total Costs Per 10 Million Population (Million I$ 2010)**	**HLYs (Million HLY) Gained Per 10 Million Population**	**ACER (I$ Per HLY)**	**ICER (I$ Per HLY) (Programmatic Expansion Path)**	**ICER (I$ Per HLY) (Health Maximizing Expansion Path)**
Current	Current scenario		337.5	6.5	52		
B295_AX95	Treatment (FLD + SLD) + Detection (Xpert + X-ray + Culture) + Drug susceptibility testing + ART prioritization for TB cases	95	525.6	7.3	72	Dominated	53279.4
B150	Treatment (FLD + SLD) + Detection (Smear + X-ray + Culture) + Drug susceptibility testing	50	251.7	4.3	58	58.0	58.0
B195	Treatment (FLD + SLD) + Detection (Smear + X-ray + Culture) + Drug susceptibility testing	95	456.7	7.3	63	69.3	69.3
B195_AX95	Treatment (FLD + SLD) + Detection (Smear + X-ray + Culture) + Drug susceptibility testing + ART prioritization for TB cases	95	486.3	7.3	66	1673.9	1673.9

Abbreviations: HLY, healthy life year; FLD, first line drugs; SLD, second line drugs; TB, tuberculosis; ACER, average cost-effectiveness ratio; ICER, incremental cost-effectiveness ratio.

**Table 17:** Costs, Effects and Cost-Effectiveness of TB Interventions in Eastern Sub-Saharan Africa Over 100 Years

	**Intervention**	**Population Coverage (%)**	**Total Costs Per 10 Million Population (Million I$ 2010)**	**HLYs (Million HLY) Gained Per 10 Million Population**	**ACER (I$ Per HLY)**	**ICER (I$ Per HLY) (Programmatic Expansion Path)**	**ICER (I$ Per HLY) (Health Maximizing Expansion Path)**
Current	Current Scenario		516	20	26		
B295	Treatment (FLD + SLD) + Detection (Xpert + X-ray + Culture) + Drug susceptibility testing	95	413	20	21	Dominated	21.7
B295_AX95	Treatment (FLD + SLD) + Detection (Xpert + X-ray + Culture) + Drug susceptibility testing + ART° prioritization for TB cases	95	431	20	21	Dominated	501.2
B150	Treatment (FLD + SLD) + Detection (Smear + X-ray + Culture) + Drug susceptibility testing	50	239	12	20	19.7	19.7
B195	Treatment (FLD + SLD) + Detection (Smear + X-ray + Culture) + Drug susceptibility testing	95	536	20	27	37.3	Dominated
B195_AX95_PX95_PXC95	Treatment (FLD + SLD) + Detection (Smear + X-ray + Culture) + Drug susceptibility testing + ART° prioritization for TB cases + Preventive therapy + Preventive therapy for children	95	614	20	30	1,306.4	15,413.7
B195_PXC95	Treatment (FLD + SLD) + Detection (Smear + X-ray + Culture) + Drug susceptibility testing + Preventive therapy for children	95	545	20	27	316.2	Dominated

Abbreviations: HLY, healthy life year; FLD, first line drugs; SLD, second line drugs; TB, tuberculosis; ACER, average cost-effectiveness ratio; ICER, incremental cost-effectiveness ratio.

**Table 18:** Costs, Effects and Cost-Effectiveness of *Plasmodium vivax *Malaria Interventions in Southeast Asia Over 100 Years

	**Intervention**	**Population Coverage (%)**	**Total Costs Per 10 Million Population (Million I$ 2010)**	**HLYs (Million HLY) Gained Per 10 Million Population**	**ACER (I$ per HLY)**	**ICER (I$ Per HLY) (Programmatic Expansion Path)**	**ICER (I$ Per HLY) (Health Maximizing Expansion Path)**	**Cases (in Million) Per 10 Million Population**	**Deaths (in Million) Per 10 Million Population**
Current	Current Scenario		2201.9	22.4	98.5	_	_	-5.87	-0.64
CMS95	Man agement of severe cases	95	146.0	21.4	6.8	6.8	6.8	0.12	-0.31
CMS50_ITN50	Management of severe cases + Insecticide-treated bed nets	50	319.3	22.2	14.4	Dominated	203.9	-5.70	-0.32
CMS80_ITN80	Management of severe cases + Insecticide-treated bed nets	80	413.9	22.4	18.4	Dominated	437.4	-5.76	-0.33
CMS95_ITN95	Management of severe cases + Insecticide-treated bed nets	95	453.9	22.5	20.2	273.5	671.4	-5.76	-0.33
CMU95_CMS95	Management of suspected uncomplicated cases + Management of severe cases	95	6412.1	22.5	284.9	Dominated	415189.9	-5.90	-0.33
CMU95_CMS95_ITN95	Management of suspected uncomplicated cases + Management of severe cases + Insecticide-treated bed nets	95	6762.9	22.5	300.5	445521.1	Dominated	-5.90	-0.33

Abbreviation: HLY, healthy life year; ACER, average cost-effectiveness ratio; ICER, incremental cost-effectiveness ratio.

**Table 19:** Costs, Effects and Cost-Effectiveness of *Plasmodium falciparum* Malaria Interventions in Eastern sub-Saharan Africa Over 100 Years

	**Intervention**	**Population Coverage (%)**	**Total Costs Per 10 Million Population (Million I$ 2010)**	**HLYs (million HLY) Gained Per 10 Million Population**	**ACER (I$ Per HLY)**	**ICER (I$ per HLY) (Programmatic Expansion Path)**	**ICER (I$ Per HLY) (Health Maximizing Expansion Path)**	**Cases Averted (in Million)Per 10 Million Population**	**Deaths Averted (in Million)Per 10 Million Population**
Current	Current scenario		1657.9	19.1	87.0			-5.0	-0.3
CMS95	Management of severe cases	95	556.8	23.3	23.9	23.9	23.9	0.5	-0.4
CMU80_CMS80_ITN80	Management of suspected uncomplicated cases + Management of severe cases + Insecticide-treated bed nets	80	3430.7	45.3	75.8	Dominated	232.2	-15.8	-0.7
CMU95_CMS95_ITN95	Management of suspected uncomplicated cases + Management of severe cases + Insecticide-treated bed nets	95	4715.8	48.5	97.3	165.2	398.9	-16.5	-0.8
CMU95_CMS95_ITN95_RTSS95	Management of suspected uncomplicated cases + Management of severe cases + Insecticide treated bed nets + Malaria vaccine with RTS,S	95	4939.3	48.6	101.6	1792.7	1792.7	-16.5	-0.8
CMU80_D_CMS80_ITN80	Management of suspected uncomplicated cases with treatment seeking fever cases RDT tested + Management of severe cases + Insecticide-treated bed nets	80	2791.8	42.5	65.7	Dominated	116.4	-15.6	-0.7

Abbreviations: HLY, healthy life year; RDT, Malaria rapid diagnostic test; ACER, average cost-effectiveness ratio; ICER, incremental cost-effectiveness ratio.

###  HIV Results

In South-East Asia, the intervention focused on FSWs at 95% coverage would be the most cost-effective intervention and is thus adopted first on the expansion path (Table 14). FSW interventions are both behavioural and biomedical. FSWs face very high risk, often the highest of any population group, and given that incidence is part of the cost-efficiency equation, FSW interventions are expected to be highly cost-effective (the more infections there are to avert, the higher the cost-effectiveness). However, it is important to note that FSWs may become hard to reach a high level of coverage given the nature of discrimination against them, and the lack of human-rights based platforms for functional intervention strategies. VMMC at 95% coverage would be the most cost-effective in eastern sub-Saharan Africa (Table 15). VMMC is an essential component of HIV prevention and is widely recognized as cost-effective in several African countries,^66,67^ our study corroborated this conclusion. VMMC is biomedical and behavioural, and also incidence reducing, and in these respects is like the FSW intervention mentioned in the previous paragraph.

In both regions, the largest HLY gains come through a combination of interventions at 95% coverage including HIV testing services, ART treatment as prevention for all HIV positive adults, children and PMTCT Option B+, mass media communication designed to increase demand and improve the use of condoms, condom provision, interventions among FSWs, PWID community outreach and peer education, youth-focused interventions and management of STIs (CB395) (Tables 14 and 15). This shows that even in concentrated epidemic settings, HIV requires a combination approach, built around ART expansion, to achieve the objectives for overall burden reduction. This means that a full suite of comprehensive approaches needs to be deployed.

Comparing the “current” intervention, at the reference time of 2010, to the expansion path: In southeast Asia, the current intervention is seen to be more cost-effective than any of the combination interventions studied in this analysis (Figure 1) and is therefore on the expansion path. Details on the expansion paths for eastern sub-Saharan Africa can be found in Figure 2.

###  Tuberculosis Results

TB treatments are known to be highly cost-effective.^7^ In both regions, the basic care-and-control scenario including treatment (FLD [first line drugs] FLD + SLD [second line drugs]), detection (Smear + X-ray + Culture) and drug susceptibility cannot be unbundled since screening cannot be implemented separately from treatment. The expansion path shows increasing levels of coverage culminating at the highest (95%) in order to achieve maximum health gains.

 Following the programmatic expansion path, and as resources become available, more interventions with lower cost-effectiveness but which are still cost-effective would be added. In eastern sub-Saharan African, where the global TB/HIV burden is high, preventive therapy for HIV-positive TB cases not on ART and on ART with LTBI, preventive therapy for children with LTBI and ART prioritization for notified HIV-positive TB cases would be progressively combined to the basic care-and-control scenario. In southeast Asia, ART prioritization for notified HIV-positive TB cases would be added to the basic care-and-control scenario.

For southeast Asia we observe that an average package of current interventions for the reference time of 2010 is on the expansion path (Figure 3), similar to the HIV results discussed above. In eastern sub-Saharan Africa, therefore, the question is not finetuning coverage levels, but rather programmatic expansion (Figure 4) since average current practice at the reference time of 2010 is on the programmatic expansion path.

###  Malaria Results

####  Plasmodium vivax Malaria Results

In South-East Asia, management of severe cases is the most cost-effective intervention (Table 18). As severe malaria is fatal in nearly all cases without treatment, successfully treating the severe cases at reasonable cost results in important health benefits in terms of averted mortality. However, promptly treating uncomplicated malaria is necessary to avoid severe cases, as well as are preventive interventions to reduce case incidence. Prevention and case management are therefore the keys to cost-effective control of malaria.

The package of average current interventions at the reference time of 2010 is on the programmatic expansion path (Figure 5).

#### Plasmodium falciparum Malaria Results

As for the *P. vivax* malaria results, above, in eastern sub-Saharan Africa, management of severe cases is highly cost-effective. Following the programmatic expansion path, management of suspected uncomplicated cases and ITN would be added to form the treatment and incidence reducing combination, which is key to cost-effective control of malaria. Malaria vaccine with RTS,S, at 95% coverage would complement the combination, adding to incidence reduction and maximizing the HLYs gained (Table 19).

The package of average coverage levels at the reference time of 2010 is well in the interior of the expansion path (Figure 6), highlighting the opportunity for efficiency gains without sacrificing programmatic criteria.

## Discussion

###  Principal Findings

 This study provides a quantitative assessment of allocative efficiency within three critical infectious-disease programme areas: HIV, TB and Malaria. By retrospectively shining a spotlight on what programme development and scaling up achieved during the first decade of the 21st century (2000-2010), it aims to assist policy-makers in understanding what worked in obtaining value for money for HIV, TB and malaria.

Over the study period, the global community has done relatively well for HIV, TB, and malaria with respect to both efficiency and programmatic criteria. The role of international assistance, financial and technical, arguably was critical to these successes. Commonly used interventions, at the reference time of 2010, for HIV, TB and malaria were cost-effective, with cost-effectiveness ratios less than I$ 100/HLY saved for virtually not only optimal interventions but for most of those included in this study. This level of cost-effectiveness would qualify interventions in the health sub-sector of HIV, TB, and malaria as “best buys” by conventional international standards such as those contained in the Appendix 3 of the Global Action Plan or the Prevention and Control of Non-Communicable Diseases, 2013-2020.^68,69^ It is essential to make this point when there is still a common perception that, for example, HIV and TB treatment regimens are prohibitively expensive compared to the health gains they offer. For southeast Asia, an average package of implemented interventions was found to be on the programmatic expansion path. In eastern sub-Saharan Africa, an average package of implemented interventions performed only slightly worse in cost-effectiveness terms than other combinations.

 Comparison of the health-maximizing expansion path versus the programmatic expansion path shows different patterns depending on the disease and the region. However, where they differ, selection of the programmatic expansion path involves some opportunity costs in health terms. These less cost-effective but programmatically superior options therefore represent choices for policy-makers: in lower- resource settings there may be arguments in favour of the health-maximizing expansion path (since the alternative represents a long-term future optimum), whereas in less constrained settings it may make sense to observe the phasing of the programmatic expansion path. Actual choices in either type of setting may of course reflect the existence of asset-specific investments that have already been made.

### Policy Implications

 As we look across the health sector, especially in low-income countries and low- and middle-income countries, coverage levels typically are far below what is required for optimal disease control and elimination. Although more needs to be done, high coverage levels have already been achieved in many countries. This means that populations in need of health services have, at least on average, the opportunity to receive many of the services they require. In addition, however, when we look at the mix of interventions being implemented, we can see that actual practice is highly cost-effective, at least on average and at aggregate level. So, not only are coverage levels higher in HIV, TB and malaria than for other conditions in low-income countries and low- and middle-income countries but also the mix of interventions that are implemented and are highly cost-effective.

 It may be hard to recognize how unusual this finding is. In almost no other area of global health is a similar case observed, at least outside of high-income countries. So, an interesting question to ask is why this has happened? Although we cannot know the answer for sure, we discuss some hypotheses that seem likely, based on our experience of performing economic evaluations across disease areas and countries during the past two decades.

 First, it seems to us that these results are not independent of the fact substantial political will was generated not only in countries but also internationally for a massive push to increase coverage with effective intervention in these three disease areas. Significant international but more importantly domestic funding was moreover mobilized. Finally, HIV, TB and malaria programmes at global level have explicitly involved the systematic use of epidemiological and economic modelling evidence in the development of their policies for nearly 20 years, initially through the Joint United Nations Programme on HIV/AIDS (UNAIDS) Reference Group on Estimates Modelling and Projections but subsequently as a practice generalized across these three programme areas. The concept of evidence-based medicine, and evidence-based policy, is strongly anchored in the culture of these three disease programme areas.

 As noted above, unprecedented levels of international donor funding and technical assistance has also arguably played a catalytic role, though the role of country financing has been larger in absolute terms. For example, the Global Fund has been able to provide a platform for international collective action independent from bilateral funding mechanisms and priorities and has also mobilized additional conditional funding for the three diseases, including from domestic sources, in the worst affected countries. This suggests the extent to which sustained collective action combined with evidence-based policies can have an impact on health outcomes even in the most resource-constrained settings.

 Major donors such as Pepfar and the Global Fund and hybrid actors such as Unitaid have likely played an important catalysing role in not only financing interventions but also in market-shaping and in ensuring the presence of high quality technical advice and the application of international normative guidance, such as from WHO technical programmes, in the worst affected countries. While we cannot demonstrate this hypothesis scientifically, it is important to recall that the funding provided by Global Fund is, in many respects, only notional, as the absolute amounts provided cannot explain the overall increase in coverage witnessed since the beginning of the MDG era. Other funders have notably been important, including bilateral funders and Pepfar. But moreover, and quite importantly, all these actors have catalysed domestic financing that has become more important as economic growth has continued in these regions.

 These observations are not a cause for complacency. Coverage levels are still inadequate from the standpoint of the economic and disease burden inflicted on the regions studied. Without continued and renewed efforts, regression to lower levels of epidemic control is inevitable and in some cases is now already being witnessed. International collective action must continue to support the case for this global public good.

### Limitations of the Analysis

Our analysis is based on the average combination of interventions used in typical countries in the studied regions in the reference year 2010. Our results are intended to be indicative of performance relative to the global knowledge of best practice at the time, rather than prescriptive packages intended for countries to implement now. In addition, some key population groups were missing in our analysis particularly for the HIV analysis where, for example, the target group for sex workers could have included men rather than just women. Transgender people or prisoners could also have been represented. These choices, while bringing greater realism, would have been challenging to model. Some other limitations to this paper are related to the methodological approach to cost-effectiveness analysis in general and the GCEA in particular and are discussed in more details elsewhere.^11,70^

## Acknowledgements

 We would like to thank our colleagues from WHO – Communicable Diseases for their review and comments on this paper.

## Ethical issues

 No ethical approval was sought as this is secondary data analysis.

## Competing interests

 Authors declare that they have no competing interests.

## Authors’ contributions

 AHR and JAL conceptualized, designed and drafted the manuscript. AHR, JAL, CP and OJTB acquired the data. AHR collated the database used and performed the cost-effectiveness analysis. AHR, JAL, CP, OJTB and EP contributed to the interpretation of the data, the edit and critical revision of the manuscript.

## Disclaimer

 AHR and EP are staff members of the WHO. The views expressed in this paper are solely the responsibility of the named authors and do not necessarily reflect the decisions or stated policy of the WHO or its Member states.

## Authors’ affiliations

^1^World Health Organization (WHO), Geneva, Switzerland. ^2^CERDI-CNRS-IRD-UCA, Clermont-Ferrand, France. ^3^University of Strathclyde, Glasgow, UK. ^4^Avenir Health, Glastonbury, CT, USA. ^5^Swiss Tropical and Public Health Institute, Basel, Switzerland. 6University of Basel, Basel, Switzerland.

## Supplementary files

Supplementary file 1. Costs, Effects and Incremental Cost-Effectiveness Per Disease, Per Region.

Supplementary file 2. Zoom in on the High-Impact Scenarios From the Cost-Effectiveness Expansion Path Figures.
