# Supplementary material for: Priority Setting in HIV, Tuberculosis, and Malaria – New Cost-Effectiveness Results From WHO-CHOICE
Source: Int J Health Policy Manag. 2021 Jan 3;10(11):678–96. doi: 10.34172/ijhpm.2020.251 (PMC9278379; doi:10.34172/ijhpm.2020.251)
Supplement: Supplementary file 1 — Costs, Effects and Incremental Cost-Effectiveness Per Disease, Per Region. [file ijhpm-10-678-s001.pdf]

Supplementary file 1. Costs, Effects And Incremental Cost-Effectiveness Per Disease, Per Region

Table S1: Costs, Effects and Cost-Effectiveness of HIV Interventions in Southeast Asia over 100 Years

	Intervention	Pop. coverage (%)	Total costs per 10 million population (million I$ 2010)	Healthy life years (million HLY) gained per 10 million population	ACER (I$ per HLY)	ICER (I$ per HLY) (Programmatic expansion path)	ICER (I$ per HLY) (Health maximizing expansion path)
Current	Current Scenario		1339.1	44.7	30	Dominated	Dominated
ART350	HIV testing services + Antiretroviral therapy for all HIV positive adults with CD4 < 350, all HIV positive children =<2 yrs, children>2 yrs with CD4 <350, pregnant women Option B+	50	4632.6	37.0	125	Dominated	Dominated
ART380	HIV testing services + Antiretroviral therapy for all HIV positive adults with CD4 < 350, all HIV positive children =<2 yrs, children>2 yrs with CD4 <350, pregnant women Option B+	80	5605.4	50.2	112	Dominated	Dominated
ART395	HIV testing services + Antiretroviral therapy for all HIV positive adults with CD4 < 350, all HIV positive children =<2 yrs,	95	6335.4	52.5	121	Dominated	Dominated

	children>2 yrs with CD4 <350, pregnant women Option B+						
ART550	HIV testing services + Antiretroviral therapy for all HIV positive adults with CD4 < 500, all HIV positive children =<2 yrs, children>2 yrs with CD4<500, pregnant women Option B+	50	4665.0	39.0	120	Dominated	Dominated
ART580	HIV testing services + Antiretroviral therapy for all HIV positive adults with CD4 < 500, all HIV positive children =<2 yrs, children>2 yrs with CD4<500, pregnant women Option B+	80	5609.5	50.8	110	Dominated	Dominated
ART595	HIV testing services + Antiretroviral therapy for all HIV positive adults with CD4 < 500, all HIV positive children =<2 yrs, children>2 yrs with CD4<500, pregnant women Option B+	95	6335.8	52.6	120	Dominated	Dominated
CB150	ART3 + MMCO + FSW +PWID + MSM +YFI + Management of Sexually	50	4111.5	48.5	85	Dominated	Dominated

	Transmitted Infections +VMMC						
CB180	ART3 + MMCO + FSW +PWID + MSM +YFI + Management of Sexually Transmitted Infections +VMMC	80	5963.1	51.5	116	Dominated	Dominated
CB195	ART3 + MMCO + FSW +PWID + MSM +YFI + Management of Sexually Transmitted Infections +VMMC	95	6878.0	52.8	130	Dominated	Dominated
CB250	ART5 + MMCO + FSW +PWID + MSM +YFI + Management of Sexually Transmitted Infections +VMMC	50	4156.6	48.8	85	Dominated	Dominated
CB280	ART5 + MMCO + FSW +PWID + MSM +YFI + Management of Sexually Transmitted Infections +VMMC	80	5984.9	51.7	116	Dominated	Dominated
CB295	ART5 + MMCO + FSW +PWID + MSM +YFI + Management of Sexually Transmitted Infections +VMMC	95	6887.6	52.9	130	Dominated	Dominated
CB350	TASP + MMCO + FSW +PWID + MSM +YFI + Management of Sexually	50	4193.6	49.2	85	Dominated	201.1

	Transmitted Infections +VMMC						
CB380	TASP + MMCO + FSW +PWID + MSM +YFI + Management of Sexually Transmitted Infections +VMMC	80	5999.3	52.0	115	Dominated	Dominated
CB395	TASP + MMCO + FSW +PWID + MSM +YFI + Management of Sexually Transmitted Infections +VMMC	95	6887.5	53.0	130	210.3	2781.0
FSW50	Female Sex Workers and clients	50	111.0	9.4	12	Dominated	Dominated
FSW80	Female Sex Workers and clients	80	119.8	16.6	7	Dominated	Dominated
FSW95	Female Sex Workers and clients	95	124.3	20.8	6	6.0	6.0
PWID50	People Who Inject Drugs community outreach and peer education	50	107.5	1.5	72	Dominated	Dominated
PWID80	People Who Inject Drugs community outreach and peer education	80	114.3	1.8	64	Dominated	Dominated
PWID95	People Who Inject Drugs community outreach and peer education	95	117.7	1.9	62	Dominated	Dominated
MMCO50	Mass Media communication designed to increase demand and improve use	50	218.2	15.7	14	Dominated	Dominated

	of COndoms, and condom provision						
MMCO80	Mass Media communication designed to increase demand and improve use of COndoms, and condom provision	80	281.6	25.4	11	Dominated	Dominated
MMCO95	Mass Media communication designed to increase demand and improve use of COndoms, and condom provision	95	313.4	29.9	10	Dominated	20.7
MSM50	Interventions targeting Men who have Sex with Men	50	135.1	0.5	277	Dominated	Dominated
MSM80	Interventions targeting Men who have Sex with Men	80	158.4	0.8	204	Dominated	Dominated
MSM95	Interventions targeting Men who have Sex with Men	95	170.1	0.9	184	Dominated	Dominated
TASP50	HIV testing services + Antiretroviral therapy Treatment AS Prevention for all HIV positive adults, children and PMTCT* Option B+	50	4649.8	41.2	113	Dominated	Dominated
TASP80	HIV testing services + Antiretroviral therapy Treatment AS Prevention for all HIV positive	80	5603.9	51.3	109	Dominated	Dominated

	adults, children and PMTCT* Option B+						
TASP95	HIV testing services + Antiretroviral therapy Treatment AS Prevention for all HIV positive adults, children and PMTCT* Option B+	95	6325.8	52.8	120	Dominated	602.5
YFI50	Youth Focused Interventions	50	242.3	0.7	328	Dominated	Dominated
YFI80	Youth Focused Interventions	80	330.0	1.2	281	Dominated	Dominated
YFI95	Youth Focused Interventions	95	373.9	1.4	269	Dominated	Dominated

Abbreviations: HLY, healthy life year; ACER, average cost-effectiveness ratio; ICER, incremental cost-effectiveness ratio; TasP, Treatment as prevention; MMCO, mass media communication and condom provision; FSW, female sex worker; PWID, people who inject drug; MSM, men who have sex with men; VMMC, voluntary medical male circumcision; YFI, youth focused intervention; PMTCT, prevention of mother-to-child transmission

Table S2: Costs, Effects and Cost-Effectiveness of HIV interventions in Eastern Sub-Saharan Africa over 100 years

	Intervention	Pop. cover age (%)	Total costs per 10 million population (million I$ 2010)	Healthy life years (million HLY) gained per 10 million populatio n	ACER (I$ per HLY)	ICER (I$ per HLY) (Programma tic expansion path)	ICER (I$ per HLY) (Health maximizing expansion path)
Current	Current Scenario		10682	513	21	Dominated	Dominated
ART350	HIV testing services + Antiretroviral therapy for all HIV positive adults with CD4 < 350, all HIV positive children =<2 yrs, children>2 yrs with CD4 <350, pregnant women Option B+	50	1379	8	165	Dominated	Dominated
ART380	HIV testing services +	80	1776	10	182	Dominated	Dominated

	Antiretroviral therapy for all HIV positive adults with CD4 < 350, all HIV positive children =<2 yrs, children>2 yrs with CD4 <350, pregnant women Option B+						
ART395	HIV testing services + Antiretroviral therapy for all HIV positive adults with CD4 < 350, all HIV positive children =<2 yrs, children>2 yrs with CD4 <350, pregnant women Option B+	95	1967	12	162	Dominated	Dominated
ART550	HIV testing services + Antiretroviral therapy for all HIV positive adults with CD4 < 500, all HIV positive children =<2 yrs, children>2 yrs with CD4<500, pregnant women Option B+	50	1436	8	170	Dominated	Dominated
ART580	HIV testing services + Antiretroviral therapy for all HIV positive adults with CD4 < 500, all HIV positive children =<2 yrs, children>2 yrs with CD4<500,	80	1814	10	174	Dominated	Dominated

	pregnant women Option B+						
ART595	HIV testing services + Antiretroviral therapy for all HIV positive adults with CD4 < 500, all HIV positive children =<2 yrs, children>2 yrs with CD4<500, pregnant women Option B+	95	1973	12	159	Dominated	Dominated
CB150	ART3 + MMCO + FSW +PWID + MSM +YFI + Management of Sexually Transmitted Infections +VMMC	50	9456	486	19	Dominated	Dominated
CB180	ART3 + MMCO + FSW +PWID + MSM +YFI + Management of Sexually Transmitted Infections +VMMC	80	8094	584	14	Dominated	Dominated
CB195	ART3 + MMCO + FSW +PWID + MSM +YFI + Management of Sexually Transmitted Infections +VMMC	95	8697	602	14	Dominated	Dominated
CB250	ART5 + MMCO + FSW +PWID + MSM +YFI + Management of Sexually Transmitted Infections +VMMC	50	10058	503	20	Dominated	Dominated

CB280	ART5 + MMCO + FSW +PWID + MSM +YFI + Management of Sexually Transmitted Infections +VMMC	80	8283	590	14	Dominated	Dominated
CB295	ART5 + MMCO + FSW +PWID + MSM +YFI + Management of Sexually Transmitted Infections +VMMC	95	8745	604	14	28.9	Dominated
CB350	TASP + MMCO + FSW +PWID + MSM +YFI + Management of Sexually Transmitted Infections +VMMC	50	1055 9	518	20	Dominated	Dominated
CB380	TASP + MMCO + FSW +PWID + MSM +YFI + Management of Sexually Transmitted Infections +VMMC	80	8386	594	14	Dominated	28.7
CB395	TASP + MMCO + FSW +PWID + MSM +YFI + Management of Sexually Transmitted Infections +VMMC	95	8781	605	15	Dominated	34.2
FSW50	Female Sex Workers and clients	50	319	31	10	Dominated	Dominated
FSW80	Female Sex Workers and clients	80	352	52	7	Dominated	Dominated

FSW95	Female Sex Workers and clients	95	369	62	6	Dominated	Dominated
PWID50	People Who Inject Drugs community outreach and peer education	50	429	2	254	Dominated	Dominated
PWID80	People Who Inject Drugs community outreach and peer education	80	474	2	250	Dominated	Dominated
PWID95	People Who Inject Drugs community outreach and peer education	95	496	2	249	Dominated	Dominated
MMCO50	Mass Media communication designed to increase demand and improve use of COndoms, and condom provision	50	624	127	5	Dominated	Dominated
MMCO80	Mass Media communication designed to increase demand and improve use of COndoms, and condom provision	80	791	238	3	Dominated	Dominated
MMCO95	Mass Media communication designed to increase demand and improve use of COndoms, and condom provision	95	886	294	3	Dominated	Dominated
MSM50	Interventions targeting Men who have Sex with Men	50	391	0	1249	Dominated	Dominated
MSM80	Interventions targeting Men who have Sex with Men	80	434	1	796	Dominated	Dominated
MSM95	Interventions targeting Men who have Sex with Men	95	456	1	673	Dominated	Dominated

VMMC50	Voluntary Medical Medical Circumcision	50	484	131		Dominated	Dominated
VMMC80	Voluntary Medical Male Circumcision	80	626	249		Dominated	Dominated
VMMC95	Voluntary Medical Male Circumcision	95	704	326		2.2	2.2
YFI50	Youth Focused Interventions	50	718	6	4	Dominated	Dominated
YFI80	Youth Focused Interventions	80	991	10	3	Dominated	Dominated
YFI95	Youth Focused Interventions	95	1127	12	2	Dominated	Dominated

Abbreviations: HLY, healthy life year; ACER, average cost-effectiveness ratio; ICER, incremental cost-effectiveness ratio; TasP, treatment as prevention; MMCO, mass media communication and condom provision; FSW, female sex worker; PWID, people who inject drug; MSM, men who have sex with men; VMMC, voluntary medical male circumcision; YFI, youth focused intervention; PMTCT, prevention of mother-to-child transmission

Table S3: Costs, Effects and Cost-Effectiveness of TB Interventions in Southeast Asia over 100 Years

	Intervention	Pop. coverage (%)	Total costs per 10 million population (million I$ 2010)	Healthy life years (million HLY) gained per 10 million population	ACER (I$ per HLY)	ICER (I$ per HLY) (Programmatic expansion path)	ICER (I$ per HLY) (Health maximizing expansion path)
Current	Current Scenario		337.5	6.5	52	Dominated	Dominated
B250	Treatment (FLD+SLD) +Detection (Xpert +X-ray+ Culture) +Drug susceptibility testing	50	417.8	4.7	89	Dominated	Dominated
B280	Treatment (FLD+SLD) +Detection (Xpert +X-ray+ Culture) +Drug susceptibility testing	80	612.0	7.3	84	Dominated	Dominated
B295	Treatment (FLD+SLD) +Detection (Xpert +X-ray+ Culture)	95	500.4	7.3	68	Dominated	Dominated

	+Drug susceptibility testing						
B250_AX50	Treatment (FLD+SLD)+Detection (Xpert + X-ray +Culture) +Drug susceptibility testing + ART° prioritization for TB cases	50	445.0	4.7	95	Dominated	Dominated
B280_AX80	Treatment (FLD+SLD)+Detection (Xpert + X-ray +Culture) +Drug susceptibility testing + ART° prioritization for TB cases	80	638.5	7.3	88	Dominated	Dominated
B295_AX95	Treatment (FLD+SLD)+Detection (Xpert + X-ray +Culture) +Drug susceptibility testing + ART° prioritization for TB cases	95	525.6	7.3	72	Dominated	53279.4
B250_AX50_PX50_PXC50	Treatment (FLD+SLD) +Detection (Xpert + X-ray +Culture) +Drug susceptibility testing + ART° prioritization for TB cases + Preventive therapy + Preventive therapy for children	50	500.4	4.8	105	Dominated	Dominated
B280_AX80_PX80_PXC80	Treatment (FLD+SLD) +Detection (Xpert + X-ray +Culture) +Drug susceptibility testing + ART° prioritization for TB cases +	80	689.7	7.3	95	Dominated	Dominated

	Preventive therapy + Preventive therapy for children						
B295_AX95_PX95_PXC95	Treatment (FLD+SLD) +Detection (Xpert + X-ray +Culture) +Drug susceptibility testing + ART° prioritization for TB cases + Preventive therapy + Preventive therapy for children	95	577.9	7.3	79	Dominated	Dominated
B250_PX50	Treatment (FLD +SLD) +Detection (Xpert + X-ray +Culture) +Drug susceptibility testing +Preventive therapy	50	444.8	4.8	93	Dominated	Dominated
B280_PX80	Treatment (FLD +SLD) +Detection (Xpert + X-ray +Culture) +Drug susceptibility testing +Preventive therapy	80	635.1	7.3	87	Dominated	Dominated
B295_PX95	Treatment (FLD +SLD) +Detection (Xpert + X-ray +Culture) +Drug susceptibility testing +Preventive therapy	95	525.0	7.3	72	Dominated	Dominated
B250_PXC50	Treatment (FLD +SLD) +Detection (Xpert + X-ray +Culture) +Drug susceptibility testing + Preventive therapy for children	50	446.5	4.7	95	Dominated	Dominated
B280_PXC80	Treatment (FLD +SLD) +Detection (Xpert + X-ray +Culture) +Drug	80	640.7	7.3	88	Dominated	Dominated

	susceptibility testing + Preventive therapy for children						
B295_PXC95	Treatment (FLD +SLD) +Detection (Xpert + X-ray +Culture) +Drug susceptibility testing + Preventive therapy for children	95	528.2	7.3	72	Dominated	Dominated
B150	Treatment (FLD +SLD) +Detection (Smear+ X-ray+ Culture) +Drug susceptibility testing	50	251.7	4.3	58	58.0	58.0
B180	Treatment (FLD +SLD) +Detection (Smear+ X-ray+ Culture) +Drug susceptibility testing	80	454.1	7.2	63	Dominated	Dominated
B195	Treatment (FLD +SLD) +Detection (Smear+ X-ray+ Culture) +Drug susceptibility testing	95	456.7	7.3	63	69.3	69.3
B150_AX50	Treatment (FLD +SLD)+Detection (Smear+ X-ray+ Culture) +Drug susceptibility testing + ART° prioritization for TB cases	50	278.8	4.3	64	Dominated	Dominated
B180_AX80	Treatment (FLD +SLD)+Detection (Smear+ X-ray+ Culture) +Drug susceptibility testing + ART° prioritization for TB cases	80	482.0	7.2	67	Dominated	Dominated
B195_AX95	Treatment (FLD +SLD)+Detection	95	486.3	7.3	66	1673.9	1673.9

	(Smear+ X-ray+ Culture) +Drug susceptibility testing + ART° prioritization for TB cases						
B150_AX50_PX50 _PXC50	Treatment (FLD +SLD) +Detection (Smear+ X-ray+ Culture) +Drug susceptibility testing + ART° prioritization for TB cases + Preventive therapy + Preventive therapy for children	50	330.1	4.4	75	Dominated	Dominated
B180_AX80_PX80 _PXC80	Treatment (FLD +SLD) +Detection (Smear+ X-ray+ Culture) +Drug susceptibility testing + ART° prioritization for TB cases + Preventive therapy + Preventive therapy for children	80	534.3	7.2	74	Dominated	Dominated
B195_AX95_PX95 _PXC95	Treatment (FLD +SLD) +Detection (Smear+ X-ray+ Culture) +Drug susceptibility testing + ART° prioritization for TB cases + Preventive therapy + Preventive therapy for children	95	538.7	7.3	74	Dominated	Dominated
B150_PX50	Treatment (FLD +SLD)+Detection (Smear+ X-ray+ Culture) +Drug susceptibility testing + Preventive therapy	50	280.0	4.4	63	Dominated	Dominated
B180_PX80	Treatment (FLD +SLD)+Detection	80	479.9	7.2	67	Dominated	Dominated

	(Smear+ X-ray+ Culture) +Drug susceptibility testing + Preventive therapy						
B195_PX95	Treatment (FLD +SLD)+Detection (Smear+ X-ray+ Culture) +Drug susceptibility testing + Preventive therapy	95	482.4	7.3	66	Dominated	Dominated
B150_PXC50	Treatment (FLD +SLD)+Detection (Smear+ X-ray+ Culture) +Drug susceptibility testing + Preventive therapy for children	50	275.3	4.3	63	Dominated	Dominated
B180_PXC80	Treatment (FLD +SLD)+Detection (Smear+ X-ray+ Culture) +Drug susceptibility testing + Preventive therapy for children	80	481.2	7.2	67	Dominated	Dominated
B195_PXC95	Treatment (FLD +SLD)+Detection (Smear+ X-ray+ Culture) +Drug susceptibility testing + Preventive therapy for children	95	484.0	7.3	66	Dominated	Dominated

Abbreviations: HLY, healthy life year; ACER, average cost-effectiveness ratio; ICER, incremental cost-effectiveness ratio; FLD, first line drugs; SLD, second line drugs; TB, tuberculosis

Table S4: Costs, Effects and Cost-Effectiveness of TB interventions in Eastern Sub-Saharan Africa over 100 years

	Intervention	Pop. coverage (%)	Total costs per 10 million population (millio	Healthy life years (million HLY) gained per 10 million	ACER (I$ per HLY)	ICER (I$ per HLY) (Programmatic expansion path)	ICER (I$ per HLY) (Health maximizing expansion path)

			n I$ 2010)	populatio n			
Current	Current Scenario		516	20	26	Dominated	Dominated
B250	Treatment (FLD+SLD) +Detection (Xpert +X-ray+ Culture) +Drug susceptibility testing	50	471	13	36	Dominated	Dominated
B280	Treatment (FLD+SLD) +Detection (Xpert +X-ray+ Culture) +Drug susceptibility testing	80	695	20	35	Dominated	Dominated
B295	Treatment (FLD+SLD) +Detection (Xpert +X-ray+ Culture) +Drug susceptibility testing	95	413	20	21	Dominated	21.7
B250_AX50	Treatment (FLD+SLD)+Detection (Xpert + X-ray +Culture) +Drug susceptibility testing + ART° prioritization for TB cases	50	503	13	38	Dominated	Dominated
B280_AX80	Treatment (FLD+SLD)+Detection (Xpert + X-ray +Culture) +Drug susceptibility testing + ART° prioritization for TB cases	80	719	20	36	Dominated	Dominated
B295_AX95	Treatment (FLD+SLD)+Detection (Xpert + X-ray +Culture) +Drug susceptibility	95	431	20	21	Dominated	501.2

	testing + ART° prioritization for TB cases						
B250_AX50_PX50_PXC50	Treatment (FLD+SLD) +Detection (Xpert + X-ray +Culture) +Drug susceptibility testing + ART° prioritization for TB cases + Preventive therapy + Preventive therapy for children	50	579	13	43	Dominated	Dominated
B280_AX80_PX80_PXC80	Treatment (FLD+SLD) +Detection (Xpert + X-ray +Culture) +Drug susceptibility testing + ART° prioritization for TB cases + Preventive therapy + Preventive therapy for children	80	787	20	39	Dominated	Dominated
B295_AX95_PX95_PXC95	Treatment (FLD+SLD) +Detection (Xpert + X-ray +Culture) +Drug susceptibility testing + ART° prioritization for TB cases + Preventive therapy + Preventive therapy for children	95	492	20	24	Dominated	Dominated
B250_PX50	Treatment (FLD +SLD) +Detection (Xpert + X-ray +Culture) +Drug susceptibility testing	50	518	13	39	Dominated	Dominated

	+Preventive therapy						
B280_PX80	Treatment (FLD +SLD) +Detection (Xpert + X-ray +Culture) +Drug susceptibility testing +Preventive therapy	80	741	20	37	Dominated	Dominated
B295_PX95	Treatment (FLD +SLD) +Detection (Xpert + X-ray +Culture) +Drug susceptibility testing +Preventive therapy	95	456	20	23	Dominated	Dominated
B250_PXC50	Treatment (FLD +SLD) +Detection (Xpert + X-ray +Culture) +Drug susceptibility testing + Preventive therapy for children	50	499	13	38	Dominated	Dominated
B280_PXC80	Treatment (FLD +SLD) +Detection (Xpert + X-ray +Culture) +Drug susceptibility testing + Preventive therapy for children	80	718	20	36	Dominated	Dominated
B295_PXC95	Treatment (FLD +SLD) +Detection (Xpert + X-ray +Culture) +Drug susceptibility testing + Preventive therapy for children	95	432	20	21	Dominated	Dominated
B150	Treatment (FLD +SLD) +Detection (Smear+ X-ray+ Culture) +Drug susceptibility testing	50	239	12	20	19.7	19.7

B180	Treatment (FLD +SLD) +Detection (Smear+ X-ray+ Culture) +Drug susceptibility testing	80	553	20	28	Dominated	Dominated
B195	Treatment (FLD +SLD) +Detection (Smear+ X-ray+ Culture) +Drug susceptibility testing	95	536	20	27	37.3	Dominated
B150_AX50	Treatment (FLD +SLD)+Detection (Smear+ X-ray+ Culture) +Drug susceptibility testing + ART° prioritization for TB cases	50	273	12	23	Dominated	Dominated
B180_AX80	Treatment (FLD +SLD)+Detection (Smear+ X-ray+ Culture) +Drug susceptibility testing + ART° prioritization for TB cases	80	582	20	29	Dominated	Dominated
B195_AX95	Treatment (FLD +SLD)+Detection (Smear+ X-ray+ Culture) +Drug susceptibility testing + ART° prioritization for TB cases	95	565	20	28	Dominated	Dominated
B150_AX50_PX50 _PXC50	Treatment (FLD +SLD) +Detection (Smear+ X-ray+ Culture) +Drug susceptibility testing + ART° prioritization for TB cases + Preventive therapy + Preventive therapy for children	50	335	12	27	Dominated	Dominated

B180_AX80_PX80_PXC80	Treatment (FLD +SLD) +Detection (Smear+ X-ray+ Culture) +Drug susceptibility testing + ART° prioritization for TB cases + Preventive therapy + Preventive therapy for children	80	658	20	33	Dominated	Dominated
B195_AX95_PX95_PXC95	Treatment (FLD +SLD) +Detection (Smear+ X-ray+ Culture) +Drug susceptibility testing + ART° prioritization for TB cases + Preventive therapy + Preventive therapy for children	95	614	20	30	1,306.4	15,413.7
B150_PX50	Treatment (FLD +SLD)+Detection (Smear+ X-ray+ Culture) +Drug susceptibility testing + Preventive therapy	50	286	12	23	Dominated	Dominated
B180_PX80	Treatment (FLD +SLD)+Detection (Smear+ X-ray+ Culture) +Drug susceptibility testing + Preventive therapy	80	605	20	30	Dominated	Dominated
B195_PX95	Treatment (FLD +SLD)+Detection (Smear+ X-ray+ Culture) +Drug susceptibility testing + Preventive therapy	95	580	20	29	Dominated	Dominated
B150_PXC50	Treatment (FLD +SLD)+Detection (Smear+ X-ray+	50	256	12	21	Dominated	Dominated

	Culture) +Drug susceptibility testing + Preventive therapy for children						
B180_PXC80	Treatment (FLD +SLD)+Detection (Smear+ X-ray+ Culture) +Drug susceptibility testing + Preventive therapy for children	80	580	20	29	Dominated	Dominated
B195_PXC95	Treatment (FLD +SLD)+Detection (Smear+ X-ray+ Culture) +Drug susceptibility testing + Preventive therapy for children	95	545	20	27	316.2	Dominated

Abbreviations: HLY, healthy life year; ACER, average cost-effectiveness ratio; ICER, incremental cost-effectiveness ratio; FLD, first line drugs; SLD, second line drugs; TB, tuberculosis

Table S5: Costs, Effects and Cost-Effectiveness of *P. vivax* Malaria Interventions in Southeast Asia over 100 years

	Intervention	Pop. coverage (%)	Total costs per 10 million population (million I$ 2010)	Healthy life years (million HLY) gained per 10 million population	ACER (I$ per HLY)	ICER (I$ per HLY) (Programmatic expansion path)	ICER (I$ per HLY) (Health maximizing expansion path)
CURRENT	Current Scenario		2,202	22	98	Dominated	Dominated
CMS50	Management of severe cases	50	121	11	11	Dominated	Dominated
CMS80	Management of severe cases	80	138	18	8	Dominated	Dominated
CMS95	Management of severe cases	95	146	21	7	6.8	6.8
CMS50_ITN50	Management of severe cases + Insecticide-treated bed nets	50	319	22	14	Dominated	203.9

CMS80_ITN80	Management of severe cases + Insecticide-treated bed nets	80	414	22	18	Dominated	437.4
CMS95_ITN95	Management of severe cases + Insecticide-treated bed nets	95	454	22	20	273.5	671.4
CMU50_CMS50	Management of suspected uncomplicated cases + Management of severe cases	50	2,048	20	104	Dominated	Dominated
CMU80_CMS80	Management of suspected uncomplicated cases + Management of severe cases	80	4,338	22	193	Dominated	Dominated
CMU95_CMS95	Management of suspected uncomplicated cases + Management of severe cases	95	6,412	23	285	Dominated	415,189.9
CMU50_CMS50_ITN50	Management of suspected uncomplicated cases + Management of severe cases + Insecticide-treated bed nets	50	2,251	22	100	Dominated	Dominated
CMU80_CMS80_ITN80	Management of suspected uncomplicated cases + Management of severe cases + Insecticide-treated bed nets	80	4,650	22	207	Dominated	Dominated
CMU95_CMS95_ITN95	Management of suspected uncomplicated cases + Management of severe cases +	95	6,763	23	300	445,521.1	Dominated

	Insecticide-treated bed nets						
CMUPQX50_CMS 50	Management of suspected uncomplicated cases + Management of severe cases (with primaquine only given to non-G6PDd° males)	50	2,085	15	143	Dominated	Dominated
CMUPQX80_CMS 80	Management of suspected uncomplicated cases + Management of severe cases (with primaquine only given to non-G6PDd° males)	80	4,341	22	194	Dominated	Dominated
CMUPQX95_CMS 95	Management of suspected uncomplicated cases + Management of severe cases (with primaquine only given to non-G6PDd° males)	95	6,413	23	285	Dominated	Dominated
CMUPQX50_CMS 50_ITN50	Management of suspected uncomplicated cases + Management of severe cases + Insecticide-treated bed nets (with primaquine only given to non-G6PDd° males)	50	2,251	22	101	Dominated	Dominated
CMUPQX80_CMS 80_ITN80	Case Management Uncomplicated with G6PD def. tests & Case Management Severe & Insecticide-treated bed nets	80	4,650	22	207	Dominated	Dominated

CMUPQX95_CMS95_ITN95	Case Management Uncomplicated with G6PD def. tests & Case Management Severe & Insecticide-treated bed nets	95	6,763	23	300	Dominated	Dominated
ITN50	Insecticide-treated bed nets	50	300	22	14	Dominated	Dominated
ITN80	Insecticide-treated bed nets	80	394	22	18	Dominated	Dominated
ITN95	Insecticide-treated bed nets	95	434	22	20	Dominated	Dominated

Abbreviations: HLY, healthy life year; ACER, average cost-effectiveness ratio; ICER, incremental cost-effectiveness ratio

Table S6: Costs, Effects and Cost-Effectiveness of *P. falciparum* Malaria Interventions in Eastern Sub-Saharan Africa over 100 years

	Intervention	Pop. coverage (%)	Total costs per 10 million population (million I$ 2010)	Healthy life years (million HLY) gained per 10 million population	ACER (I$ per HLY)	ICER (I$ per HLY) (Programmatic expansion path)	ICER (I$ per HLY) (Health maximizing expansion path)
CURRENT	Current Scenario		1,658	19	87	Dominated	Dominated
CMS50	Management of severe cases	50	430	12	35	Dominated	Dominated
CMS80	Management of severe cases	80	514	20	26	Dominated	Dominated
CMS95	Management of severe cases	95	557	23	24	23.9	23.9
CMS50_ITN50	Management of severe cases + Insecticide-treated bed nets	50	730	8	96	Dominated	Dominated
CMS80_ITN80	Management of severe cases + Insecticide-treated bed nets	80	926	16	59	Dominated	Dominated
CMS95_ITN95	Management of severe cases +	95	1,014	20	50	Dominated	Dominated

	Insecticide-treated bed nets						
CMS50_ITN50_RTSS50	Management of severe cases + Insecticide treated bed nets + Malaria vaccine with RTS,S	50	867	7	116	Dominated	Dominated
CMS80_ITN80_RTSS80	Management of severe cases + Insecticide treated bed nets + Malaria vaccine with RTS,S	80	1,116	16	70	Dominated	Dominated
CMS95_ITN95_RTSS95	Management of severe cases + Insecticide treated bed nets + Malaria vaccine with RTS,S	95	1,231	20	61	Dominated	Dominated
CMS50_RTSS50	Management of severe cases + Malaria vaccine with RTS, S	50	567	12	46	Dominated	Dominated
CMS80_RTSS80	Case Management Severe & RTSS malaria vaccine	80	706	20	36	Dominated	Dominated
CMS95_RTSS95	Case Management Severe & RTSS malaria vaccine	95	773	24	33	Dominated	Dominated
CMU50_CMS50	Management of suspected uncomplicated cases + Management of severe cases	50	1,817	24	74	Dominated	Dominated
CMU80_CMS80	Management of suspected uncomplicated cases + Management of severe cases	80	3,077	40	77	Dominated	Dominated
CMU95_CMS95	Management of suspected uncomplicated cases + Management of severe cases	95	4,317	46	94	Dominated	Dominated

CMU50_CMS50_ITN50	Management of suspected uncomplicated cases + Management of severe cases + Insecticide-treated bed nets	50	2,056	35	59	Dominated	Dominated
CMU80_CMS80_ITN80	Management of suspected uncomplicated cases + Management of severe cases + Insecticide-treated bed nets	80	3,431	45	76	Dominated	232.2
CMU95_CMS95_ITN95	Management of suspected uncomplicated cases + Management of severe cases + Insecticide-treated bed nets	95	4,716	48	97	165.2	398.9
CMU50_CMS50_ITN50_RTSS50	Management of suspected uncomplicated cases + Management of severe cases + Insecticide treated bed nets + Malaria vaccine with RTS,S	50	2,194	35	63	Dominated	Dominated
CMU80_CMS80_ITN80_RTSS80	Management of suspected uncomplicated cases + Management of severe cases + Insecticide treated bed nets + Malaria vaccine with RTS,S	80	3,625	45	80	Dominated	Dominated
CMU95_CMS95_ITN95_RTSS95	Management of suspected uncomplicated cases +	95	4,939	49	102	1,792.7	1,792.7

	Management of severe cases + Insecticide treated bed nets + Malaria vaccine with RTS,S						
CMU50_CMS50_ RTSS50	Management of suspected uncomplicated cases + Management of severe cases + Malaria vaccine with RTS, S	50	1,956	25	78	Dominated	Dominated
CMU80_CMS80_ RTSS80	Management of suspected uncomplicated cases + Management of severe cases + Malaria vaccine with RTS, S	80	3,272	40	82	Dominated	Dominated
CMU95_CMS95_ RTSS95	Management of suspected uncomplicated cases + Management of severe cases + Malaria vaccine with RTS, S	95	4,378	46	95	Dominated	Dominated
CMU50_D_CMS5 0	Management of suspected uncomplicated cases + Management of severe cases (but treatment seeking fever cases RDT° tested)	50	2,105	21	99	Dominated	Dominated
CMU80_D_CMS8 0	Management of suspected uncomplicated cases + Management of severe cases (but treatment seeking fever cases RDT° tested)	80	3,283	32	104	Dominated	Dominated

CMU95_D_CMS95	Management of suspected uncomplicated cases + Management of severe cases (but treatment seeking fever cases RDT° tested)	95	4,341	42	103	Dominated	Dominated
CMU50_D_CMS50_ITN50	Management of suspected uncomplicated cases + Management of severe cases + Insecticide-treated bed nets (but treatment seeking fever cases RDT° tested)	50	2,137	32	67	Dominated	Dominated
CMU80_D_CMS80_ITN80	Management of suspected uncomplicated cases + Management of severe cases + Insecticide-treated bed nets (but treatment seeking fever cases RDT° tested)	80	2,792	43	66	Dominated	116.4
CMU95_D_CMS95_ITN95	Management of suspected uncomplicated cases + Management of severe cases + Insecticide-treated bed nets (but treatment seeking fever cases RDT° tested)	95	4,421	46	96	Dominated	Dominated
CMU50_D_CMS50_ITN50_RTSS50	Management of suspected uncomplicated cases + Management of severe cases +	50	2,271	32	71	Dominated	Dominated

	Insecticide treated bed nets + Malaria vaccine with RTS,S (but treatment seeking fever cases RDT° tested)						
CMU80_D_CMS80_ITN80_RTSS80	Management of suspected uncomplicated cases + Management of severe cases + Insecticide treated bed nets + Malaria vaccine with RTS,S (but treatment seeking fever cases RDT° tested)	80	3,512	43	82	Dominated	Dominated
CMU95_D_CMS95_ITN95_RTSS95	Management of suspected uncomplicated cases + Management of severe cases + Insecticide treated bed nets + Malaria vaccine with RTS,S (but treatment seeking fever cases RDT° tested)	95	4,635	46	100	Dominated	Dominated
CMU50_D_CMS50_RTSS50	Management of suspected uncomplicated cases + Management of severe cases + Malaria vaccine with RTS, S (but treatment seeking fever cases RDT° tested)	50	2,241	21	105	Dominated	Dominated
CMU80_D_CMS80_RTSS80	Management of suspected uncomplicated cases +	80	3,476	34	102	Dominated	Dominated

	Management of severe cases + Malaria vaccine with RTS, S (but treatment seeking fever cases RDT° tested)						
CMU95_D_CMS95_RTSS95	Management of suspected uncomplicated cases + Management of severe cases + Malaria vaccine with RTS, S (but treatment seeking fever cases RDT° tested)	95	4,327	42	102	Dominated	Dominated

Abbreviations: HLY, healthy life year; ACER, average cost-effectiveness ratio; ICER, incremental cost-effectiveness ratio; RDT, Malaria rapid diagnostic test
