# Supplementary material for: Priority Setting in HIV, Tuberculosis, and Malaria – New Cost-Effectiveness Results From WHO-CHOICE
Source: Int J Health Policy Manag. 2021 Jan 3;10(11):678–96. doi: 10.34172/ijhpm.2020.251 (PMC9278379; doi:10.34172/ijhpm.2020.251)
Supplement: Supplementary file 2 — Zoom in on the High-Impact Scenarios From the Cost-Effectiveness Expansion Path Figures. [file ijhpm-10-678-s002.pdf]

Supplementary file 2. Zoom in on the High-Impact scenarios from the Cost-Effectiveness Expansion Path Figures

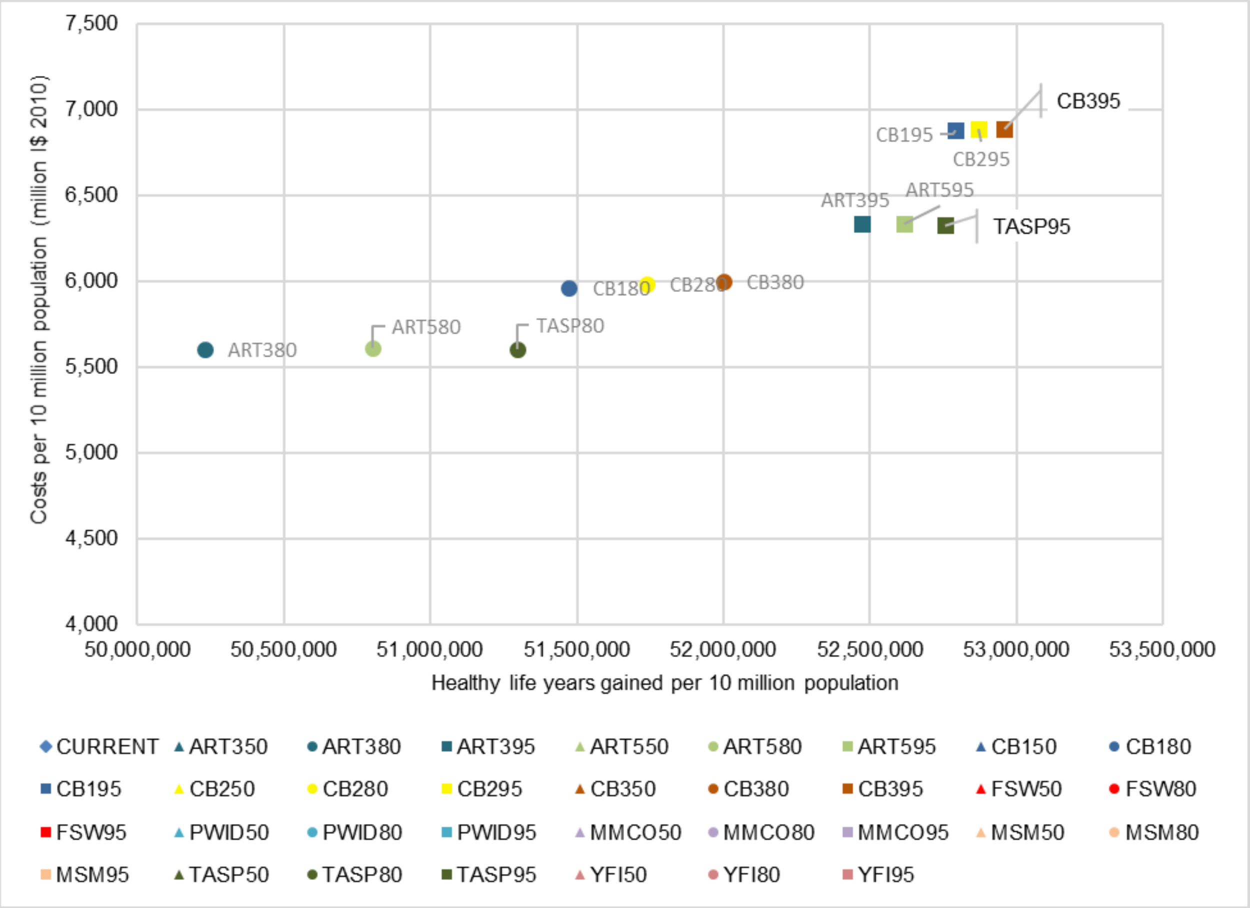

Figure S1: Zoom in on the high-impact scenarios (upper right) from the cost-effectiveness expansion path figure for HIV interventions in Southeast Asia

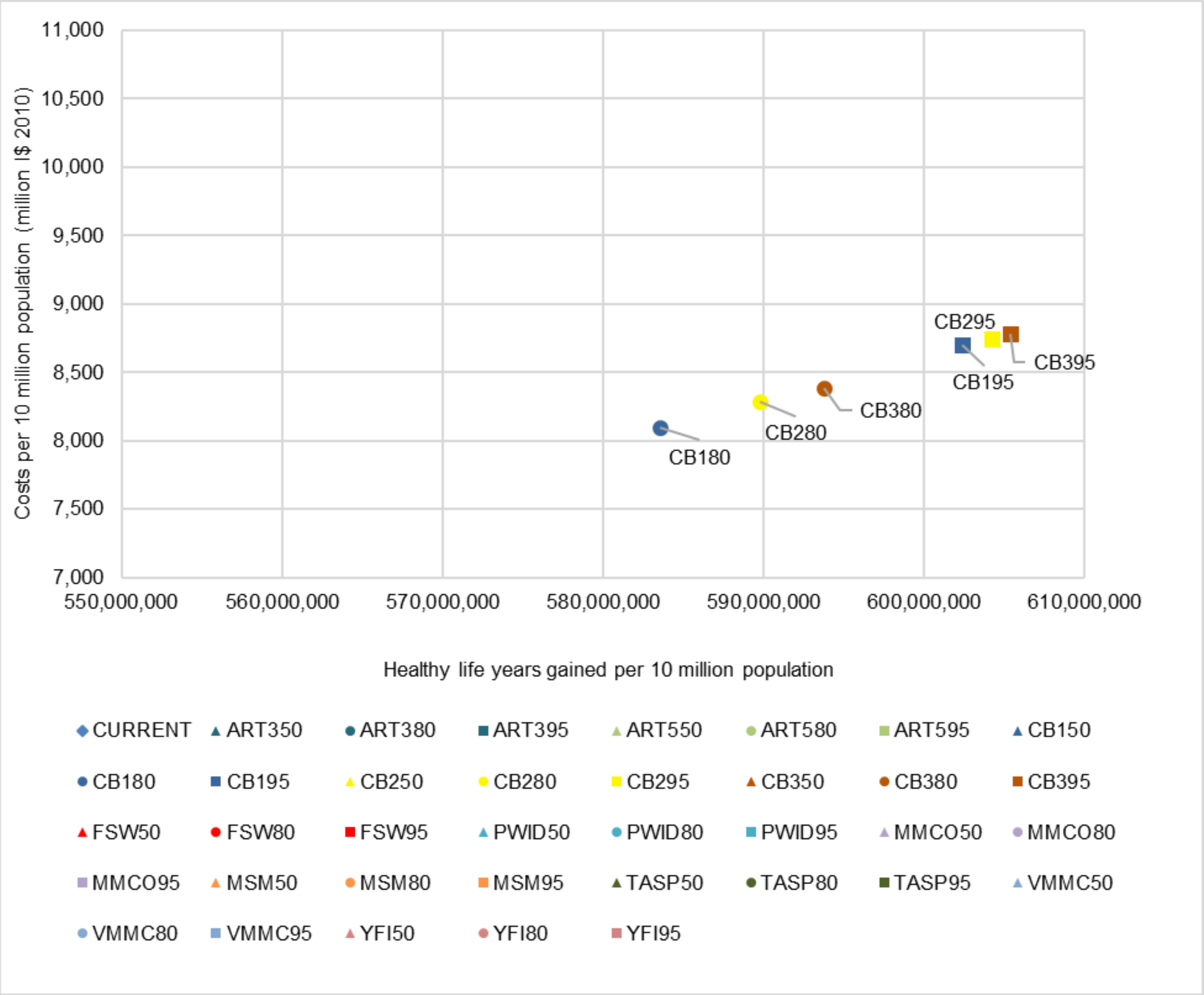

Figure S2: Zoom in on the high-impact scenarios (upper right) from the cost-effectiveness expansion path figure for HIV interventions in eastern sub-Saharan Africa

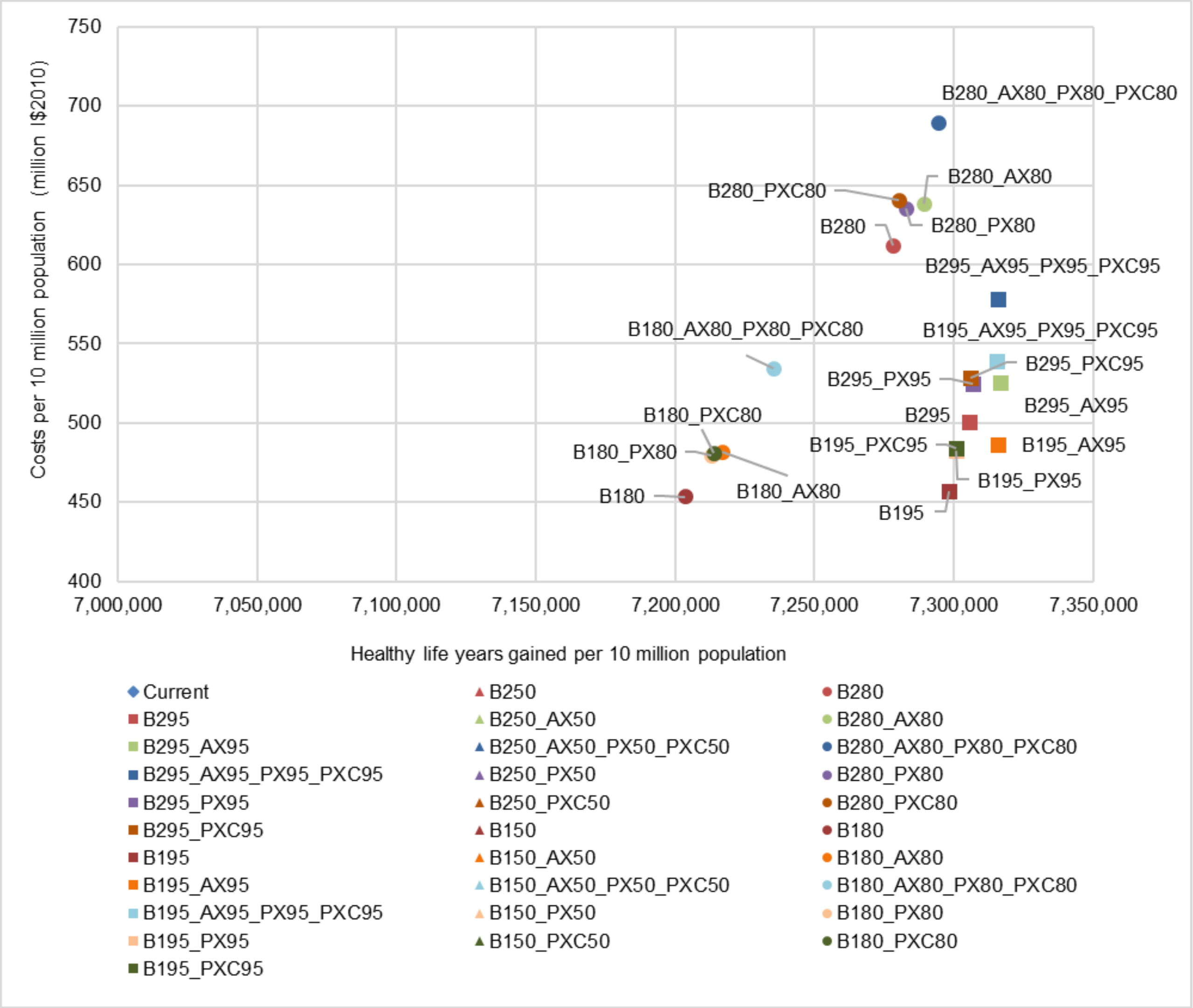

Figure S3: Zoom in on the high-impact scenarios (upper right) from the cost-effectiveness expansion path figure for TB interventions in Southeast Asia

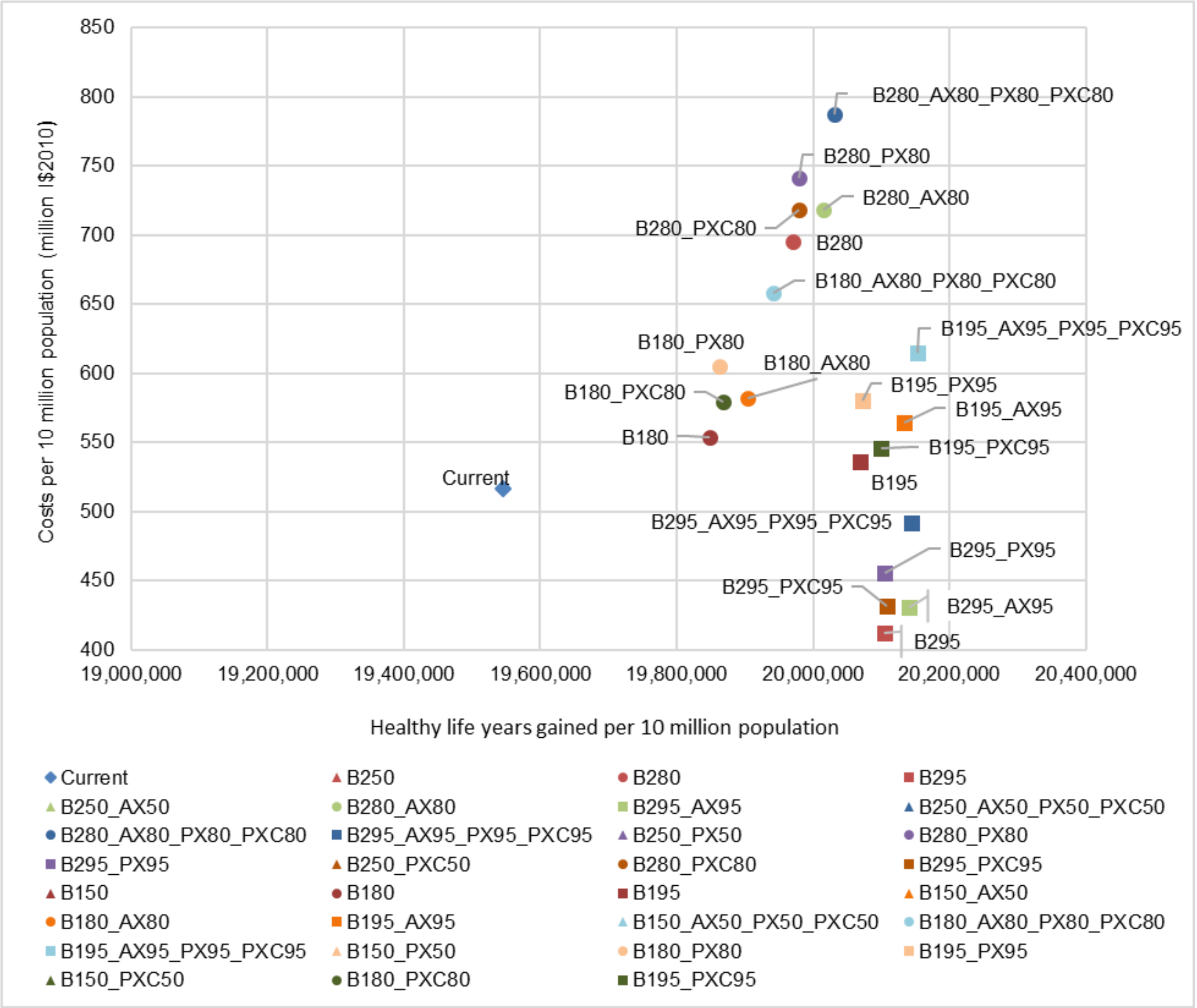

Figure S4: Zoom in on the high-impact scenarios (upper right) from the cost-effectiveness expansion path figure for TB interventions in eastern sub-Saharan Africa

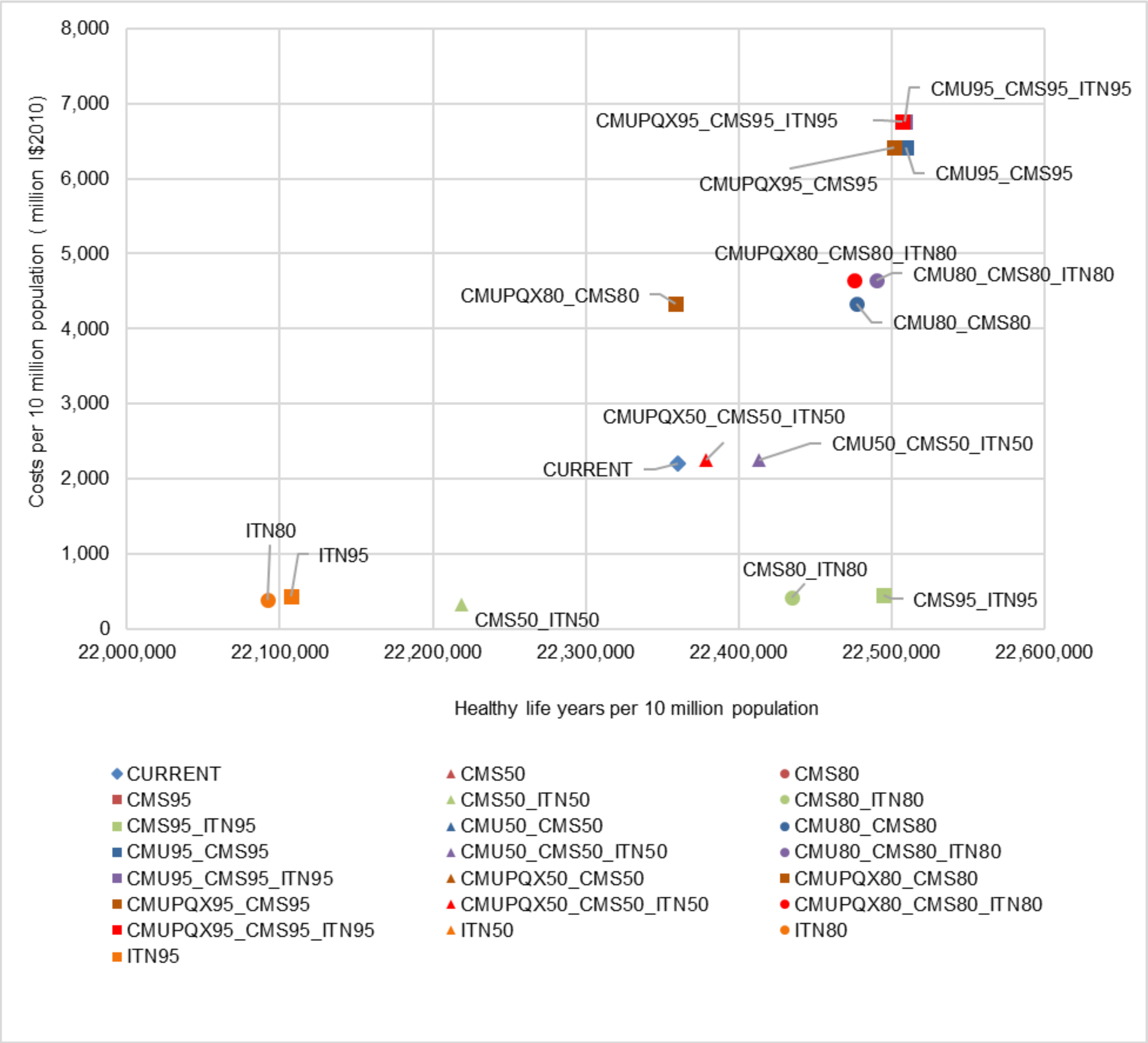

Figure S5: Zoom in on the high-impact scenarios (upper right) from the cost-effectiveness expansion path figure for *P. vivax* malaria interventions in Southeast Asia

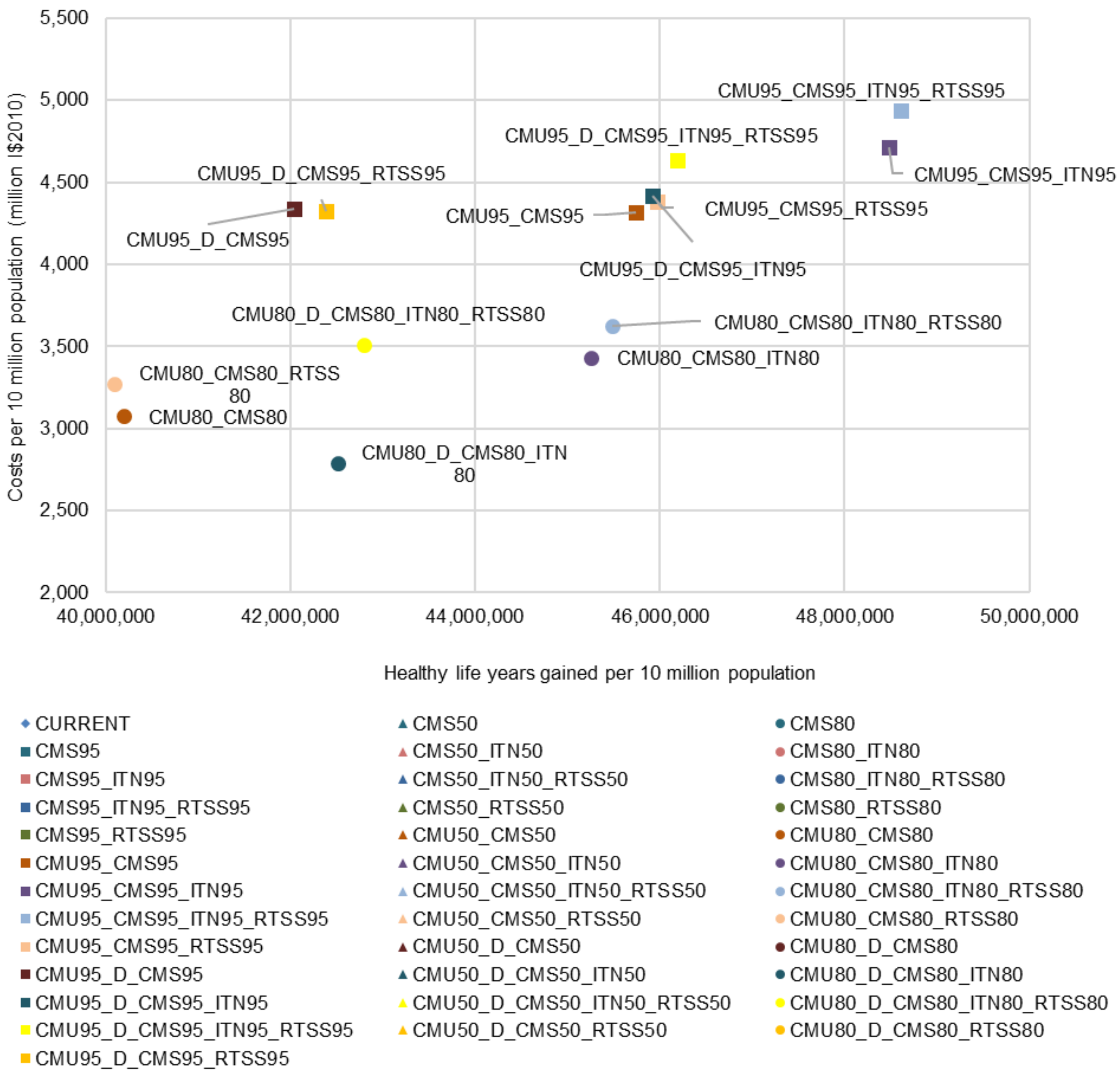

Figure S6: Zoom in on the high-impact scenarios (upper right) from the cost-effectiveness expansion path figure for *P. falciparum* malaria interventions in eastern sub-Saharan Africa
